# Stable isotopes reveal patterns of diet and mobility in the last Neandertals and first modern humans in Europe

**DOI:** 10.1038/s41598-019-41033-3

**Published:** 2019-03-14

**Authors:** Christoph Wißing, Hélène Rougier, Chris Baumann, Alexander Comeyne, Isabelle Crevecoeur, Dorothée G. Drucker, Sabine Gaudzinski-Windheuser, Mietje Germonpré, Asier Gómez-Olivencia, Johannes Krause, Tim Matthies, Yuichi I. Naito, Cosimo Posth, Patrick Semal, Martin Street, Hervé Bocherens

**Affiliations:** 1 Department of Geosciences, Biogeology, University of Tübingen, Hölderlinstrasse 12, 72074 Tübingen, Germany; 2 Department of Anthropology, California State University Northridge, 18111 Nordhoff St., Northridge, CA 91330-8244 California USA; 3 Institute for Archaeological Sciences, University of Tübingen, Rümelin Strasse 23, 72070 Tübingen, Germany; 4 Operational Direction “Earth and History of Life”, Royal Belgian Institute of Natural Sciences, Brussels, Belgium; 5 UMR 5199 PACEA, CNRS, Université de Bordeaux, Pessac, Cedex France; 6 Senckenberg Centre for Human Evolution and Palaeoenvironment (HEP) an der Universität Tübingen, Tübingen, Germany; 7 Monrepos Archaeological Research Centre for Human Behavioural Evolution, Römisch-Germanisches Zentralmuseum, Leibniz-Research Institute for Archaeology, 56567 Neuwied, Germany; 8 Institute of Ancient Studies, Johannes Gutenberg-University, University Mainz, Mainz, Germany; 9 Departmento de Estratigrafía y Paleontología, Facultad de Ciencia y Tecnología, Euskal Herriko Unibertsitatea (UPV/EHU), Barrio Sarriena s/n, 48940 Leioa, Spain; 10 IKERBASQUE, Basque Foundation for Science, Bilbao, Spain; 11 UMR 7194 CNRS, Département de Préhistoire, Muséum national d’Histoire naturelle, Musée de l’Homme, 17 Place du Trocadéro, 75016 Paris, France; 12 Centro UCM-ISCIII de Investigación sobre Evolución y Comportamiento Humanos, Avda. Monforte de Lemos 5 (Pabellón 14), 28029 Madrid, Spain; 13 Max Planck Institute for the Science of Human History, Khalaische Strasse 10, 07745 Jena, Germany; 14 Operational Direction “Scientific Service of Heritage“Royal Belgian Institute of Natural Sciences, Brussels, Belgium

## Abstract

Correlating cultural, technological and ecological aspects of both Upper Pleistocene modern humans (UPMHs) and Neandertals provides a useful approach for achieving robust predictions about what makes us human. Here we present ecological information for a period of special relevance in human evolution, the time of replacement of Neandertals by modern humans during the Late Pleistocene in Europe. Using the stable isotopic approach, we shed light on aspects of diet and mobility of the late Neandertals and UPMHs from the cave sites of the Troisième caverne of Goyet and Spy in Belgium. We demonstrate that their diet was essentially similar, relying on the same terrestrial herbivores, whereas mobility strategies indicate considerable differences between Neandertal groups, as well as in comparison to UPMHs. Our results indicate that UPMHs exploited their environment to a greater extent than Neandertals and support the hypothesis that UPMHs had a substantial impact not only on the population dynamics of large mammals but also on the whole structure of the ecosystem since their initial arrival in Europe.

## Introduction

**Figure 1:**
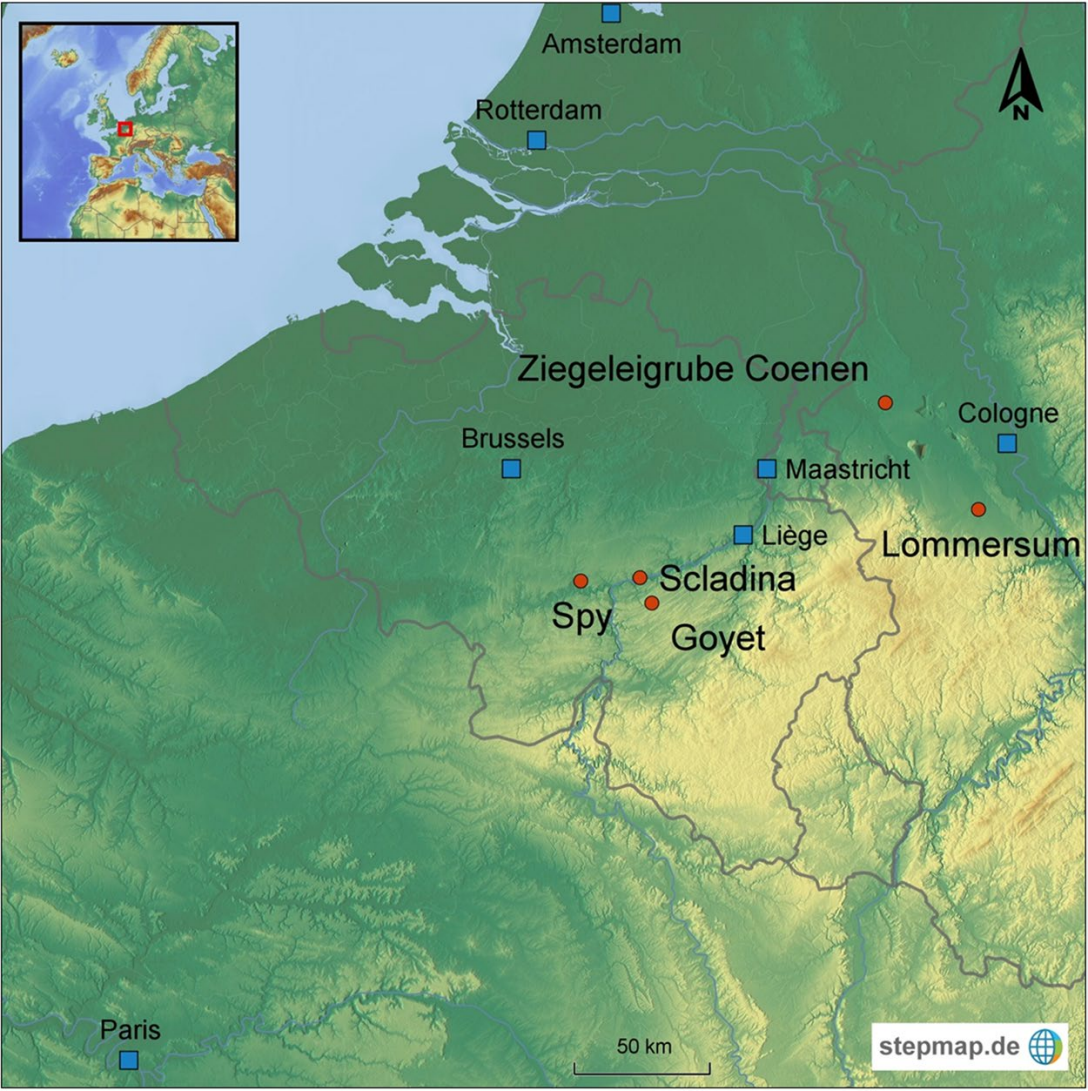
Current map of Europe with the site locations (red dots). Map produced through the website stepmap.de.

Nowadays modern humans (*Homo sapiens*) are the only species of humans left on Earth. This was different during the Late Pleistocene when Neandertals and Upper Pleistocene modern humans (UPMH) coexisted in Europe. Relatively soon after the arrival of UPMHs in this region about 45–43,000 years ago, the Neandertals became extinct^1–4^. Differences in the ecological niches of UPMHs and Neandertals while coexisting in the same ecosystems are regularly suggested as being the possible cause for the demise of Neandertals. Emphasis is placed on late Neandertals being ecologically less flexible than UPMHs (but see^5–7^) and therefore giving an advantage to UPMHs. According to this hypothesis, UPMHs had a broader dietary ecological spectrum, especially having possibly included more aquatic resources than that of Neandertals. This was suggested based on higher nitrogen isotopic ratios (*δ*^15^N) of bone collagen for UPMHs in comparison with late Neandertals^8–10^, with the latter supposedly having had a diet whose protein part consisted purely of terrestrial herbivorous mammal meat^11–14^. These conclusions are extremely relevant when considering overall human evolution, since they present potential causes for the extinction of Neandertals and the rise of UPMHs. Unfortunately, they are essentially drawn from only a few UPMH remains^8,15–17^ from locations where late Neandertals have not been discovered and therefore direct comparisons of the two human groups in the same ecological conditions are missing^18–22^. In addition, most of these studies are missing the environmental context of the isotopic data they present (see discussion in^12,14^). So far, a direct comparison of isotopic values of Neandertals and UPMHs through time and space has been only tentative and does not lead to reliable conclusions about potential differences in the ecological niches of the two types of humans. It is indeed crucial to contextualize human isotopic data using coeval herbivorous prey, as well as carnivorous species, since shifts of the isotopic baseline occurred for *δ*^15^N through time and space during the Late Pleistocene in Europe^23–25^. As a consequence, consuming the same diet at different time periods might lead to different nitrogen isotopic values, which would result in incorrect and misleading conclusions about the dietary strategies of humans^23,26^. The Troisième caverne of Goyet site (Belgium) provides a unique opportunity to fill the gap because skeletal remains of both types of humans representing several individuals have been recovered, directly radiocarbon dated, and their taxonomic attribution confirmed by palaeogenetic analysis^27,28^ (Fig. 1 and Supplementary Data 1).

The Goyet human remains were associated with a rich faunal assemblage, and both the human and faunal skeletal specimens are biochemically well preserved. These circumstances make this site a key location for the understanding of the ecological behavior of the last Neandertals and UPMHs close to the time of replacement between 45,000 and 35,000 years BP in Europe^1,29^. Here we applied a Bayesian mixing model (SIAR)^30^ using carbon and nitrogen stable isotopic data of bone collagen to determine the relative proportions of different prey species in the diet of the UPMHs (this study) and Neandertals from the Troisième caverne of Goyet^14^. Additionally, we used the same approach with the already published isotopic data of the late Neandertals from the nearby site of Spy^31^ in order to make regional comparisons between late Neandertal groups.

Through insights into dietary and mobility aspects of late Neandertals and UPMHs, we aim to reveal ecological aspects in the context of the transition phase from the Neandertal to UPMH occupation of Northern Europe^32^. Finally, a key assertion resulting from this study is an estimation of the ecological impact that each type of human had on the ecosystem. For this we considered faunal remains from the sites of Troisème caverne of Goyet, Spy and Scladina, contemporaneous with the Neandertals occupation of Belgium approximately 39,000 to 47,000 years ago (Supplementary Data 1, 2 3 and 4) as well as some Upper Palaeolithic (UP) sites resulting from the presence of UPMHs in Germany. We present new isotopic data from the Aurignacian open air site of Lommersum (Supplementary Data 5), which is dated to 33,250 and 35,100 years BP^33^, and which is in the vicinity of the Belgian sites (Fig. 1). This site is of high relevance in terms of providing adequate quantities of well-preserved faunal remains dating to the early UP in this region and therefore gives an insight into the fauna exploited by UPMHs. The oldest site considered here, Ziegeleigrube Coenen (ZC) in Germany (Fig. 1), is contemporary with late Neandertals and reflects the niche partitioning of the mammoth steppe fauna during a cold spell between a minimum age of ~40,000 BP to a finite age of ~47,000 BP^24,34^.

Given that mobility is a key variable, especially in relation to how interactions within the ecosystem occurred in hunter-gatherer societies^35–39^, we also investigate the mobility history using sulphur isotopic composition in bone collagen within and across two Neandertal groups, one from Spy and the other from Goyet, as well as from the UPMHs from Goyet (Tables 1, S1). In the context of evaluating potential group mobility aspects, it is of special interest that the Goyet Neandertals show features of intensive cannibalism on their highly fragmented bones, this being in contrast to the Spy Neandertals^27^. In addition to the humans, broad selections of mammal species from the Belgian sites as well as from Lommersum in Germany (Tables S2 and S3) were investigated for their sulphur stable isotopic signal. The sulphur stable isotopic composition has been found to reflect underlying geology and therefore can be considered as an indicator of geographic location with the potential to track aspects of mobility, and thus of the behavioral patterns across the landscape^40–43^.

**Table 1:** List of the stable isotopic data and related ^14^C dates from Late Pleistocene Neandertal and UPMH remains from the Troisième caverne of Goyet and Spy.

ID	Species	Lab#	^14^C age (BP)	*δ*^13^C	*δ*^15^N	*δ*^34^S	Reference for ^14^C	Reference for *δ*^13^C and *δ* ^15^N
Site: Goyet								
Q116-1	*Homo sapiens*	GrA-46175	30,880 + 170, −160	−19.1	10.9	8.6	^28^	*
Q376-3	*Homo sapiens*	GrA-60034	29,370 + 180, −170	−18.8	11.4	4.4	^28^	*
C5-1	*Homo neanderthalensis*			−19.7	12.1	10.3		*
Q48-1	*Homo neanderthalensis*			−19.6	11.3	11.5		*
Q53-4	*Homo neanderthalensis*	GrA-54022	39,870 + 400, −350	−19.0	11.7	9.7	^27^	14
Q55-1	*Homo neanderthalensis*	GrA-54257	37,860 + 350 −310	−19.2	11.3	9.8	^27^	14
Q55-4	*Homo neanderthalensis*			−19.2	11.6	11.4		14
Q56-1	*Homo neanderthalensis*	GrA-46170	38,440 + 340, −300	−19.5	11.5	9.2	^27^	14
Q57-1	*Homo neanderthalensis*	GrA-46173	41,200 + 500, −410	−19.2	11.8	10.9	^27^	14
Q57-2	*Homo neanderthalensis*	GrA-54024	36,590 + 300, −270	−19.1	11.9	10.8	^4^	14
Q57-3	*Homo neanderthalensis*	GrA-60019	38,260 + 350, −310	−19.6	11.2	10.9	^27^	14
Q 119-2	*Homo neanderthalensis*	—	—	−19.3	11.5	11.9		*
Q305-4	*Homo neanderthalensis*	GrA-46176	40,690 + 480, −400	−19.4	10.7	7.5	^27^	14
Q305-7	*Homo neanderthalensis*	—	—	−19.0	11.3	11.3		14
Q374a-1	*Homo neanderthalensis*	—	—	−19.1	11.8	10.2		14
Q376-1	*Homo neanderthalensis*	GrA-46178	39,140 + 390, −340	−19.2	10.9		^27^	14
Q376-20	*Homo neanderthalensis*	GrA-60018	37,250 + 320, −280	−19.4	11.8	11.6	^27^	14
Q376-9	*Homo neanderthalensis*	—	—	−19.2	11.8	12.9		*
Q376-25	*Homo neanderthalensis*	—	—	−19.0	11.5	11.4		*
2878-2D	*Homo neanderthalensis*	GrA-54028	32,190 + 200, −190	−19.0	12.5		^27^	*
Site: Spy								
Spy 94a (Spy II?)	*Homo neanderthalensis*	GrA-32623	35,810 + 260, −240	−19.4	11.4	3.6	^79^	31
Spy 430a (Spy II?)	*Homo neanderthalensis*	GrA-32630	33,940 + 220, −210	−20.3	10.8		^79^	31
Spy 92b (Spy I?)	*Homo neanderthalensis*	GrA-32626	36,350 + 310,−280	−19.8	10.9		^79^	31
Spy 572a (Spy I/II?)	*Homo neanderthalensis*	GrA-21546	31,810 + 250,−250	−19.8	11.0		^79^	31
Spy 646a Spy (VI)	*Homo neanderthalensis*	GrA-32627	32,970 + 200,−190	−19.8	12.5	2.6	78	*

Abbreviations: *this study. *δ*^34^S values are all from this study; note that *δ*^34^S values of samples Q48-1, Q119-2, Q305-7, Q376-9 and Q376-25  are not considered due to preservation issues^47,76,85,93^.

Thanks to the rich archeological and human fossil records of the Troisième caverne of Goyet^27,44^, our study using stable isotopes has the potential to contribute essential new information and to provide an extensive view on ecological issues of both Neandertals and UPMHs during a particularly important time in European prehistory.

## Results

### Isotopic results

For all considered samples the collagen preservation fulfilled the conditions for reliable biogenic stable carbon, nitrogen and sulphur isotopic values^45–47^ (Table 1, Tables S1, S2, S3 and Supplementary Data 6).

### Carbon and nitrogen stable isotope values

For the study we analysed collagen from two UPMHs and 18 Neandertal specimens, including six new samples from the Troisième caverne of Goyet as well as one Neandertal individual from Spy (Spy 646a) for *δ*^13^C and *δ*^15^N (Table 1, Table S1). The UPMHs yielded *δ*^13^C values of −19.1‰ for the individual represented by Q116-1 and −18.8‰ for the second individual represented by Q376-3. The *δ*^15^N values were 10.9‰ for Q116-1 and 11.4‰ for Q376-3. The Goyet Neandertals (n = 18) from this study yielded *δ*^15^N values ranging from 10.9‰ to 12.5‰ (av. 11.8‰; s.d. 0.43‰) and *δ*^13^C ranging from −19.7‰ to −19.0‰ (av. 19.2‰; s.d. 0.22‰). This study presents one Neandertal immature individual Spy 646a (Spy VI) in addition to the already published specimens representing the adults Spy I and II. The Spy Neandertal (Spy 646a) produced values of 12.5‰ for *δ*^15^N and −19.8‰ for *δ*^13^C. These additional values match those previously presented^14^.

From the site of Lommersum in Germany we analysed *δ*^13^C and *δ*^15^N ratios of collagen for horse (*Equus ferus*, n = 9), reindeer (*Rangifer tarandus*, n = 10), mammoth (*Mammuthus primigenius*, n = 1), wolf (*Canis lupus*, n = 1) and cave lion (*Panthera spelaea*, n = 1) (Table S3). Here the *δ*^13^C values obtained ranged from −20.9‰ for a horse (Lom-20) to −18.3‰ for a reindeer (Lom-8) (av. −19.7‰; s.d. 0.9‰). The *δ*^15^N values ranged from 2.0‰ for a reindeer (Lom-7) to 8.5‰ for the cave lion (Lom-15) (av. 4.8‰; s.d. 2.0‰).

### Sulphur stable isotopes

**Figure 2:**
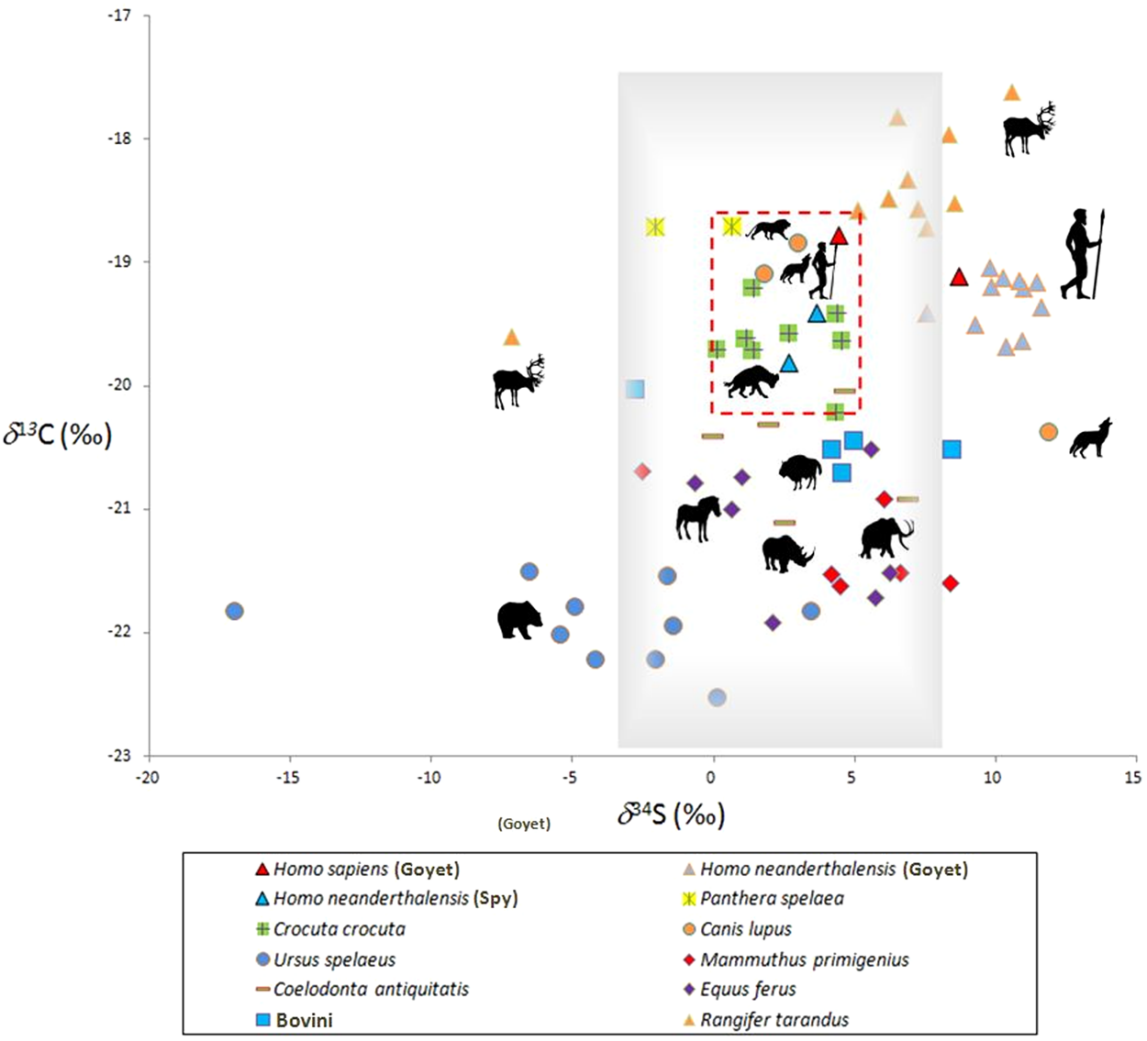
Bivariate plot of *δ*^34^S and *δ*^13^C stable isotopic values of bone collagen representing the Late Pleistocene ecosystem in Belgium. The plot includes Neandertals as well as UPMHs from Goyet and Neandertals from Spy. Faunal remains are from Scladina, Spy and Goyet. Different species are associated with different symbols and colors. Individual specimens are plotted. The shaded grey rectangle represents the local sulphur signal, the red dashed line essentially encompasses most of the carnivorous species. Carnivores reflect the average *δ*^34^S values of their prey^75^.

The *δ*^34^S values for the UPMHs were 8.6‰ (Q116-1) and 4.4‰ (Q376-3). The values for the Goyet Neandertals (n = 11) range from 7.5‰ (Q305-4) to 11.6‰ (Q376-20) with a mean of 10.2‰. The *δ*^34^S values for the Spy Neandertals were 3.6‰ for the adult Spy 94a and 2.6‰ for Spy 646a (Spy VI). The faunal *δ*^34^S values from Goyet (n = 27) range from −7.2 to 8.4‰ (mean 1.2‰; s.d. 4.1‰) and those from Scladina (n = 23) from −17.0‰ to 11.8‰ (mean 2.4‰; s.d. 5.8‰). The *δ*^34^S analysis of the Spy horse provided a value of 5.5‰ (sample IV2A 4207) (Table S2). For the Belgian sites, *δ*^34^S values were obtained from the same collagen samples as for the *δ*^13^C and *δ*^15^N data that were processed by Bocherens *et al*. and Wißing *et al*.^14,31,48,49^ or in this study. The faunal remains from Lommersum (n = 7) provided *δ*^34^S values between 2.0 and 4.7‰ (mean 3.4‰; s.d. 1.5‰) (Table S3). For Lommersum, all stable isotopes were measured on the same collagen samples. Figure 2 shows a bivariate plot of the *δ*^34^S and *δ*^13^C values from the Late Pleistocene in Belgium. The faunal remains have a mean *δ*^34^S value of 2.61‰ and the grey background in Fig. 2 represents the mean ±2 s.d. range in *δ*^34^S values of between −2.6‰ and 7.8‰.

### Reconstruction of isospace

**Figure 3:**
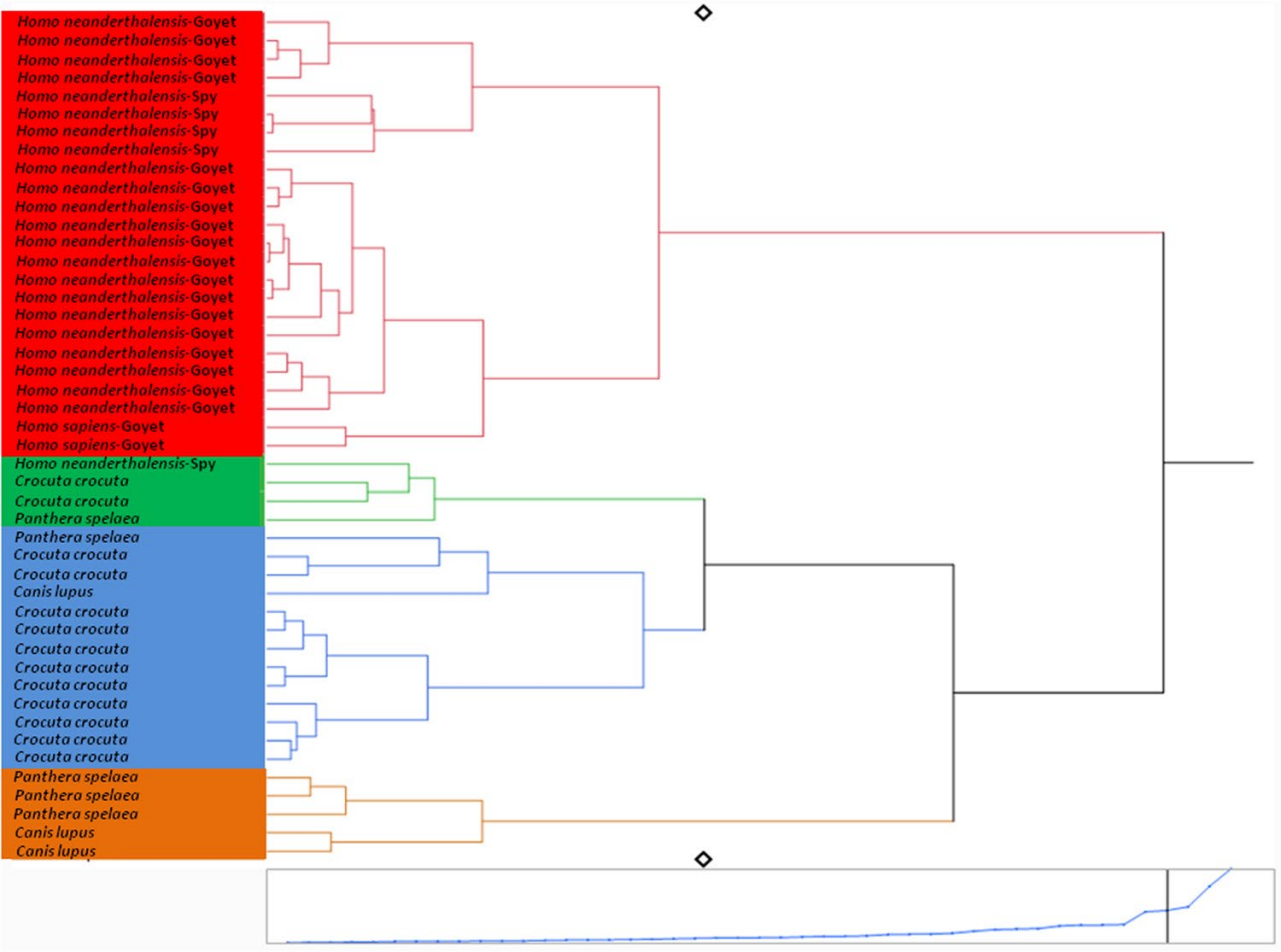
Cluster analysis showing Late Pleistocene carnivorous and omnivorous mammal species (incl. humans) from the sites of the Troisième caverne of Goyet, Scladina and Spy based on *δ*^15^N and *δ*^13^C bone collagen values. Cluster analysis using the Ward’s minimum variance method with the software SAS JMP version 10.0.

**Figure 4:**
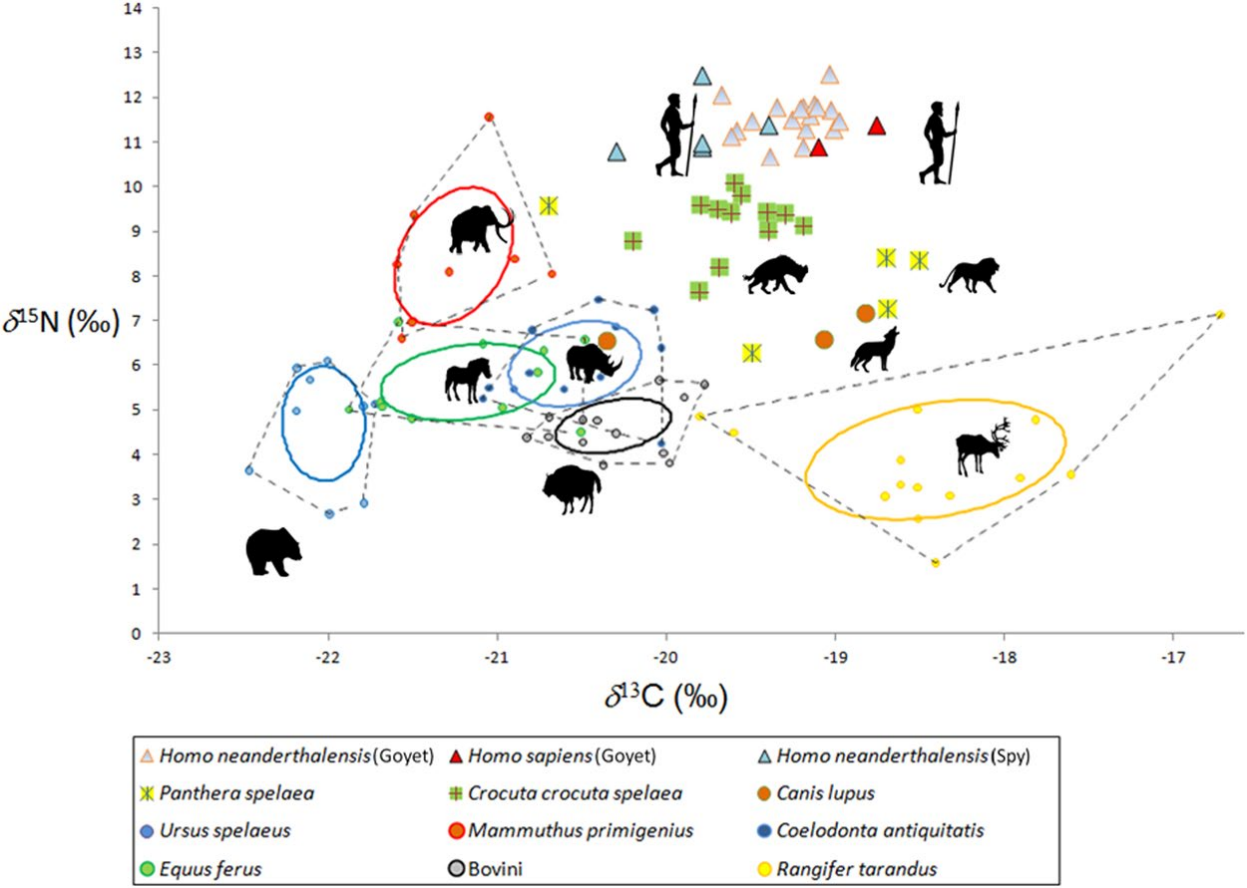
Bivariate plot of *δ*^13^C and *δ*^15^N stable isotopic values of bone collagen, representing the Late Pleistocene ecosystem from Scladina, Spy and Goyet in Belgium. The plot includes Neandertals and UPMHs from Goyet as well as Neandertals from Spy. Herbivores’ core niches (= standard ellipse areas) in total niche (dashed convex hulls) were calculated using SIBER^86^ (Stable Isotope Bayesian Ellipses in R). Omnivores and carnivores are displayed as individual specimens. Silhouettes represent species attribution. The same silhouette is used to represent Neandertals and UPMHs.

We define the isospace as the range of isotopic values typical of a given species and representing the specific ecological niche of this species in terms of the *δ*^13^C and *δ*^15^N values of its bone collagen. It is not limited to defining only the physical space, although the place of habitation impacts the isotopic signal. In our concept, the most paramount aspect is the diet, more precisely the protein part of the diet of a particular species. Isospaces can be described in several ways and we used two approaches: on the one hand a cluster analysis for the carnivorous species (Fig. 3) and the core niche concept (implemented as a classical bivariate scatter plot of *δ*^13^C and *δ*^15^N values) for the herbivorous species (Fig. 4). The cluster analysis provides information at the individual level, whereas for the herbivores the core niche concept presents the isospace at a species level (Figs. 3 and 4). The cluster analysis includes carnivorous and omnivorous species alongside the three groups of humans: the Neandertals from Spy and from the Troisième caverne of Goyet, as well as the UPMHs from Goyet. It demonstrates that the humans are the predators that isotopically overlap the least with other potential animal competitors (Fig. 3).

It is particularly interesting that among the carnivores and omnivores, the first branch-off divides the humans (except one from Spy) from all other species. All other carnivorous species group closer to each other than to Neandertals and UPMHs. This early branch-off of the humans demonstrates rather that Neandertals and UPMHs ate more similar protein sources compared to the other carnivores and omnivores. This isotopic pattern further indicates that both types of humans occupied the most distinct ecological niche among the carnivores and omnivores.

Most observable is that the different herbivorous species occupied distinct isospaces similar to the data already published in the existing literature^23,50–52^.

## Dietary Protein Reconstruction of the Goyet UPMHs

**Figure 5:**
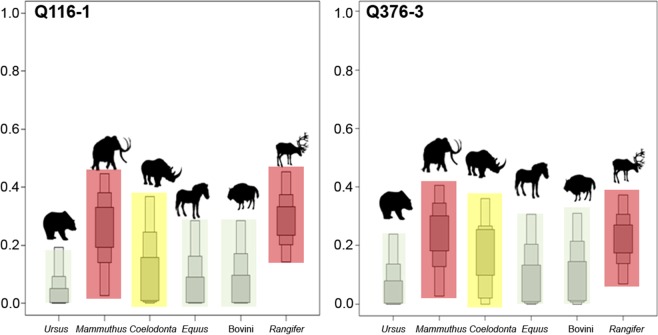
Box plots of the relative contributions (in %) of different prey species to the protein portion of the diet of the UPMHs from Goyet. Calculations were performed through the application of a Bayesian method (SIAR V4, Stable Isotope Analysis-package in R)^87,88^. Within the proportion box plots, three shades of grey are shown. Light grey represents a probability of 95%, medium grey 75% and dark grey 25%. Background colors highlight relative importance with red: most important, yellow: second most important and bright green: least important for a single species. The relative contributions of each species are similar for both individuals (Q116-1 and Q376-3). Here, faunal remains from the sites of Goyet, Scladina and Spy are included Table S1).

In this study, the relative contributions of different prey species as a dietary protein source have been reconstructed. Protein reconstruction for the Goyet Neandertals was previously presented in another study^14^. The additional Neandertal remains that are analysed here (Table 1) are very similar to those of the former study and will not be detailed here, but it is worth mentioning that the most important prey species are mammoth and reindeer for all of the analysed Neandertal remains^14^. For the UPMHs, a reconstruction of the relative contributions of the most important prey species is presented in Fig. 5 (Supplementary Figs. 2, 3 and Data 7 ).

For both UPMH individuals, the two most relevant prey species are the mammoth and the reindeer. Each species comprised roughly 25–30% of the meat protein source. The rhinoceros contributed ca. 15 to 20%, the bovines and horses around 10% of the dietary proteins. Cave bears played the least important role, with a maximum contribution of around 5% of the total protein intake. These results are similar to those of Neandertals, which indicates that both UPMHs and Neandertals had a similar prey choice (Supplementary Data 7) with preference for mammoth and reindeer.

## Discussion

**Figure 6:**
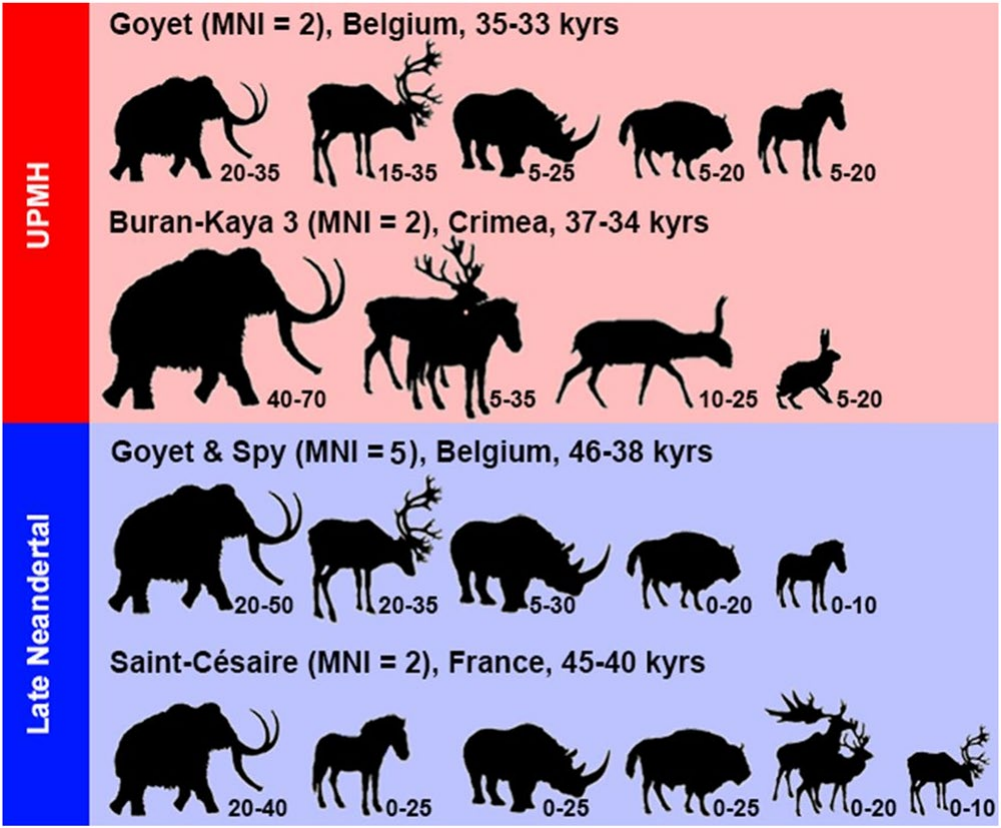
Relative proportions (in %) of different prey species to the protein intake of UPMHs and late Neandertals. Calculations based on *δ*^13^C and *δ*^15^N in percentage, using the SIAR Bayesian model (SIAR V4, Stable Isotope Analysis-package in R)^87,88^. Note that mammoth is the most important prey species, contributing systematically a minimum of ca. 20% of the dietary protein in both types of humans.

The isotopic signatures of the Goyet UPMHs and of the Neandertals are similar (Figs. 3 and 4), both indicating a purely terrestrial diet and a similar preference for particular terrestrial herbivores. The notion that UPMHs had a broader dietary spectrum cannot be supported in this study, more specifically there was no indication of intake of aquatic resources, as suggested in some studies based on relatively higher *δ*^15^N values for UPMHs^5,6,12^. The present study did not find high *δ*^15^N values, which may have indicated a substantial intake of aquatic resources. In contrast, the focus on preying upon terrestrial herbivores by UPMHs^53^ as well as by late Middle Palaeolithic humans is well documented^11,14,18,31^ and is well confirmed here for both the Goyet UPMHs and late Neandertals from Goyet and Spy (Figs. 5 and 6). The zooarchaeological records from Goyet and Spy fully support mammoth hunting episodes with a special preference for younger individuals and possibly their mothers (Supplementary Fig. 10, Supplementary Data 2, Tables S4, S5, S6,)^54^. Interestingly, based on stable isotopes, the mammoth seems to contribute the major part of the dietary protein of humans in a time range between 50,000 and 30,000 years ago and across wide areas spanning from SW France^11^ to the Crimean Peninsula^53^ (Fig. 6, Supplementary Fig. 5–8).

Analysing the bulk collagen fraction underestimates the plant protein contribution to the diet^12^, but another approach more sensitive to plant food intake using *δ*^15^N values of specific amino acids of bone collagen from Neandertals from Spy in Belgium indicates a substantial amount of plant protein in the diet of the Spy Neandertals^55,56^. This supports rather broader subsistence strategies for late Neandertals than previously considered in a palaeoecological context typical of the MIS 3. It has been argued that Neandertals altered their diets in response to changing palaeoecological conditions, while the diets of UPMHs were more associated to changes in their technological complexes, possibly having given them advantages over Neandertals^57,58^. Despite higher numbers of individuals and of analysed bone specimens for the Belgian Neandertals, we could not identify a more restricted dietary strategy in comparison to the UPMHs from Goyet. More data from other sites and areas would help to see if this is a generalized pattern or a more localized phenomenon.

We are aware that even if the relative proportions of dietary components were similar for late Neandertals and UPMHs, it is still possible that differences existed in hunting impact via exploitation intensity. This translates into a different impact on the surrounding environment, especially on the prey abundance caused by a potentially substantial human population increase during the Neandertal to UPMHs transition in Europe around 40,000 years ago^43,59,60^. Changes in isotopic niche partitioning among herbivorous prey species have been attributed to a decline of the mammoth population and the spread of the horse in the under-occupied niche^43^. Based on the finding that both UPMHs and Neandertals had a preference for mammoth meat, as demonstrated in this study and elsewhere^61,62^, a more detailed investigation into human-ecosystem interactions should offer crucial knowledge about the ecological role Neandertals and UPMHs played. Consequently, we investigated the ecological setting on a chronological scale spanning from around 45,000 to 25,000 years ago in the broader region of Western Europe, representing sites contemporaneous with late Neandertals and UPMHs (Supplementary Fig. 9). The ecological niche partitioning and especially deviations from the expected niche partitioning can reflect ecological stress, which may be recorded in the stable isotopic composition^50,63^. The mammoth was a key prey species (Fig. 6) so the potential ecological stress on it shall be investigated.

The oldest site considered, Ziegeleigrube Coenen (ZC) in Germany (Fig. 1, Supplementary Fig. 9), reflects the niche partitioning of the mammoth steppe fauna during a cold spell between a minimum age of ~40,000 years BP and a finite age of ~47,000 years BP^24,34,50,51,63^ which makes the site contemporaneous with late Neandertals. In this region, there was obviously a coherent food web structure in which all species maintained their niche, without any signs of ecological stress.

During all the following chronological phases (Supplementary Fig. 9) represented by the last Neandertal sites from Belgium and the early UP site of Lommersum as well as the Swabian Jura UP sites in Germany^43,64^, the situation changed. We observe a trend for single horse individuals to overlap in their isotopic values with those of the mammoth. During the UP in the Swabian Jura, we can even detect that individual horses interfered with the core niche of the mammoth (Supplementary Fig. 9). This trend is reinforced with the early UP which can also be seen in the total reduction of the isotopic distance of the horse and mammoth core niches (Supplementary Fig. 9, Data 8).

Not all horses during the final MP and early UP in the region studied (Fig. 1) occupied their traditional ecological niche, as observed in the context of earlier phases of mammoth steppe in Europe and other areas in Northern Eurasia and Beringia during the Late Pleistocene^14,48,49,51,52^. While considering the partial overlap of the isotopic signatures of the mammoth and horse, the SIAR model cannot create a sufficient distinction between the relative proportions of dietary protein for the UPMHs from the Troisième caverne of Goyet. An underestimation of the contribution of the horse can affect the results provided by SIAR. However, the UPMHs relied on one of the two species or perhaps both occupying this specific ecological niche. Consequently, the ecological reliance of both types of humans as predators on this niche was comparable from a qualitative point of view. We hypothesize that with the arrival of UPMHs in the study area, the predation pressure on the mammoth population increased relative to the time before. An UPMH population density up to ten times higher than during the Neandertal occupation^60,65–69^, in combination with different spatial and information exchange systems^70–73^, supports the phenomenon of the partially occupied ecological mammoth niche and its partial invasion by horses. In terms of dietary ecology, Neandertals and UPMHs behaved very similarly with respect to their prey choice, with the difference appearing to be that UPMHs exploited their resources more intensively than Neandertals and thereby causing ecological stress on the mammoth populations in the research area.

In addition to dietary ecology, facets of individual and group mobility (more precisely land use procurement strategies) are necessary to consider to be able to discuss the possible differences between late Neandertal and UPMH ecological niches^39,74,75^. Sulphur isotopic data can provide information about these aspects^76^. Figure 2 shows *δ*^34^S vs. *δ*^13^C of Late Pleistocene faunal and human remains from the Troisième caverne of Goyet, Scladina and Spy sites. To provide a representation of the local region (grey background), we used the mean values ±2 s.d. from the fauna. In comparison to all available studies presenting sulphur isotopic data from a Late Pleistocene archeological context^24,41–43^ the indicated range of around 10.0‰ in this study is the widest. However, since the geological bedrock in this area is quite diverse^77^, a wider range of sulphur isotopic compositions for the (semi-) local terrestrial ecosystem is to be expected. The defined *δ*^34^S range in this area is especially substantiated by the central positioning of most of the carnivores (the red dashed rectangle), which reflects the average *δ*^34^S values of their prey. Two carnivores, one cave lion and one canid, are outside the central area, which indicates a regular intake of protein sources with a sulphur isotopic signal different from the rest of the carnivores. Potential prey species represented by a comparable sulphur signal are the cave bear (for the cave lion) as well as the reindeer (for the canid). In Fig. 2, the Spy Neandertals plot centrally within the local signal, in between the local carnivores. Consequently, we find that the adult individual represented by Spy 94a had its main foraging area in the surrounding ecosystem, or at least in the same regions as the carnivores of the three Belgian sites. Spy 646a (Spy VI) is a child of around 1.5 years^78^, who was probably raised in this region. The sulphur values of both individuals are very close. On the other hand, the Neandertals from the Troisième caverne of Goyet yielded high *δ*^34^S values. No other species except the already mentioned canid and some reindeer yielded similar values. The *δ*^34^S values for the Goyet Neandertals clearly indicate an origin (for the main part of their diet) outside of the local ecosystem as reflected by the animal remains deposited in the Belgian caves of Spy, Scladina and Goyet. It seems unlikely that the *δ*^34^S values of the Goyet Neandertals were affected by a possible regular intake of aquatic resources, since the carbon and nitrogen isotopic values are very homogeneous for all three groups of humans. In the context of potential intake of aquatic resources, it becomes evident that the occurrence of individual faunal specimens with higher *δ*^34^S than the main group demonstrates the presence of one or more isotopically different terrestrial ecosystems that were accessible to the Goyet Neandertals and UPMHs. Unfortunately, at this stage of research we have been unable to locate the Goyet Neandertals’ catchment area but we are able to at least exclude some regions where *δ*^34^S values for Late Pleistocene faunal remains are available from. The values for Lommersum are substantially lower (Table S2), the same being true for Ziegeleigrube Coenen^24^, the Swabian Jura (SW Germany)^43^ farther south, as well as areas farther east, such as Kraków Spadzista (Southern Poland)^42^ and Predmostí I in the Moravian Plains in the Czech Republic^41^. The only known Late Pleistocene ecosystem with very similar *δ*^34^S values has been observed in SW France^43^. We therefore conclude that the Neandertals from the Troisième caverne of Goyet were not local in respect to their foraging area based on sulphur isotopic values, which is in contrast to the Neandertals from Spy whose *δ*^34^S values are consistent with the local sulphur isotopic signal.

Interestingly, the non-local Neandertals from the Troisième caverne of Goyet show evidence of intensive cannibalism^27^, which is not the case for the local Spy Neandertals^79^. These new isotopic results encourage hypothesizing that the Neandertal group from the Troisième caverne of Goyet was of foreign origin and may have been slaughtered perhaps by local inhabitants (exocannibalism), either Neandertals or another so far unknown group of older UPMHs. We can also envisage the scenario that the Goyet Neandertal group was killed somewhere else and their remains brought into the site. It is also crucial to emphasize the occurrence of early UPMH sites with ages in the range of the Goyet Neandertals in the broader region such as in Italy^3^, Germany^71^, and Austria^4^, but no Upper Palaeolithic stone tool industry with a similar age to the Neandertals from Goyet has been identified in Belgium so far. No more hypotheses may be formulated at this point, since the Goyet site has yielded the victims of cannibalistic activity and not the initiators.

The Goyet UPMHs are represented by two individuals^28^. Individual Q376-3 gave values centered in the area defined as the local *δ*^34^S signal, whereas individual Q116-1 is outside of the local *δ*^34^S signal and within the variation of the Goyet Neandertals (Fig. 2). The argument, that UPMHs had a higher intake of aquatic resources in their diet as a the reason for their *δ*^34^S values to be different (at least for one individual) to the Goyet Neandertals could only hold true if this was in combination with higher *δ*^15^N and/or *δ*^13^C values (compared to the Goyet Neandertals). The two UPMH individuals cover the total range of sulphur values (around 4‰) of all the Goyet Neandertal specimens studied (n = 11). Unfortunately, given the limited UPMH fossil record, we cannot determine if this range represents the endpoints of this chronogroup of UPMH nor if individual mobility history among UPMHs is actually more diverse than seems to be the case for the Goyet Neandertals. If the latter is true, more variable, broader and probably stronger interconnections with (trans-) regional networks, not only for land use procurement and exploitation strategies but also for the exchange of ideas and even people (see e.g. in^80^), would be supported for UPMHs. Regardless of this hypothesis, the application of a sulphur isotopic approach appears to be an adequate tool for retrieving information of spatial aspects among humans in Late Pleistocene archeological contexts. A broader dietary spectrum for UPMHs cannot be stated as being a substantial reason for the success of one human type over the other; instead, it seems necessary to investigate further the possibility of different concepts of landscape utilization that could have given UPMHs the edge over Neandertals.

## Methods

### Collagen preparation and isotopic analysis

Bone sampling followed standard procedure and a protocol modified from Longin^81^ was implemented^48^. A preliminary determination of the potential collagen preservation (nitrogen content in whole bone) was conducted^82–84^. These measurements were performed with a Vario EL III elemental analyser (Elementar) (mean standard error 0.02%, 0.05%, and 0.03% for %C, %N and %S, respectively). Isotopic measurements were performed at the Geochemical Unit of the Department of Geosciences at the University of Tübingen (Germany), using an elemental analyser NC 2500 connected to a Thermo Quest Delta + XL mass spectrometer. Collagen preservation was determined and general criteria were considered for the chemical integrity of this protein^45^. The isotopic ratios are expressed using the “*δ*” (delta) value as follows:

$${\delta }^{13}{\rm{C}}=[{(}^{13}{\rm{C}}{/}^{12}{\rm{C}})\,{\rm{sample}}/{(}^{13}{\rm{C}}{/}^{12}{\rm{C}})\,{\rm{reference}}-\,1]\times 1000\,\textperthousand ,$$

$${\delta }^{15}{\rm{N}}=[{(}^{15}{\rm{N}}{/}^{14}{\rm{N}})\,{\rm{sample}}/{(}^{15}{\rm{N}}{/}^{14}{\rm{N}})\,{\rm{reference}}-\,1]\times 1000\textperthousand ,$$

and

$${\delta }^{34}{\rm{S}}=[{(}^{34}{\rm{S}}{/}^{32}{\rm{S}})\,{\rm{sample}}/{(}^{34}{\rm{S}}{/}^{32}{\rm{S}})\,{\rm{reference}}-\,1]\times 1000\textperthousand .$$

The standard for *δ*^13^C is the internationally defined marine carbonate V-PDB. For *δ*^15^N the standard atmospheric nitrogen (AIR) is used. Samples are calibrated to *δ*^13^C values of USGS 24 (*δ*^13^C = −16.00‰, relative to V-PDB) and to *δ*^15^N values of IAEA-N-2 (*δ*^15^N = 20.30‰, relative to ATM).

The standard for *δ*^34^S is the internationally defined Vienna-Canyon Diablo Troilite (VCDT). *δ*^34^S values are calibrated to *δ*^34^S values of NBS 123 (*δ*^34^S = 17.10‰, relative to CDT), NBS 127 (*δ*^34^S = 20.31‰, relative to VCDT) and IAEA-S-1 (*δ*^34^S = −0.30‰, relative to CDT) and IAEA-S-3 (*δ*^34^S = 21.70‰, relative to CDT).

Analytical error based on laboratory standards is ±0.1‰ for *δ*^13^C values, ±0.2‰ for *δ*^15^N results and ±0.4‰ for *δ*^34^S measurements.

*δ*^34^S values with the atomic C/S_coll_ and N/S_coll_ ratios in the range of 300 to 900 and 100 to 300, respectively, were considered to be valid for our purpose^47,76,85^. Modern day mammal collagen contains sulphur from 0.14 to 0.33%^85^, which fits with the theoretical range of 0.14 to 0.29% based on DNA and amino acid sequencing^47^. Only samples with sulphur content in collagen between 0.13 and 0.24% were considered in this study.

### Cluster analysis and calculation of core niches (SIBER)

To identify patterns for individual distribution of single herbivorous and carnivorous specimens regardless of species attribution, we performed a cluster analysis using the Ward’s minimum variance method with the software SAS JMP version 10.0 (Fig. 3 and Supplementary Fig. 1). The reconstruction of the ecological niches for the herbivorous species (Fig. 4 and Supplementary Fig. 9) were done through SIBER (Stable Isotope Bayesian Ellipses in R)^86^. Through this R package, we reconstructed the complete niches (=convex hulls) and the core niches (=standard ellipse areas). The complete niche includes all individuals of a niche, but is quite sensitive to sample size. However, the core niche depicts the centre of a niche that is calculated by using most likelihood estimation and Bayesian statistics, and is less sensitive to the sample size^86^.

Here, the relative distances between species and the relative size and potential overlapping of convex hull and core niches are of special interest.

### Protein source reconstruction with the Bayesian mixing model (SIAR)

With SIAR^87,88^, a package of the software R, it is possible to estimate quantitative and qualitative aspects of the animal proteins consumed by a specific carnivore or omnivore species. For the reconstruction of a hominin diet, it is of importance to realize that the importance of plant proteins is in general underestimated. The reason for this is the existence of a non-linear isotopic correlation between the most extreme end points of a pure vegetarian and a pure carnivorous feeding behavior. Even a very small amount of meat consumption substantially increases the *δ*^15^N values of bone collagen. For example, an amount of ~50% of plant intake results in collagen *δ*^15^N values that are not even 1 standard deviation lower than the *δ*^15^N values of a pure carnivore^12^. To consider this adequately, we provide data relative to the protein source of the different prey species but not absolute values. The applied SIAR package^87,88^ in the R software version 3.3.0^88^ has the capability to cope with multiple dietary sources and incorporates uncertainties (standard deviations) in the input data. The tool provides not only a probability range for a specific protein source proportion but also the relative probability distribution at different amounts (Supplementary Figs. 2 and 7). Within the box plot generated (Fig. 5) the light grey represents a probability range of 95%, medium grey 75% and dark grey 25% for different prey proportions. The software provides diagnostic matrix plots in which the statistical dependencies between different protein sources are summarized (Supplementary Figs. 3 and 6). Dependencies have either a negative or positive character. This means, that a given protein source, e.g. A, can be partly replaced by source B if we included source C. The mixing of two or more protein sources can end up in the isotopic range of the third source. The SIAR software takes into account these correlations among different sources (prey species), which end up in an increasing probability range. In correspondence to previous works^89–92^, we applied a Trophic Enrichment Factor (TEF) of +1.1 ± 0.2‰ for *δ*^13^C and +3.8 ± 1.1‰ for *δ*^15^N values. The only precondition here is that we assumed that the prey species are the herbivorous ones we included in our study.

## Supplementary information

Supplementary Dataset
