# Supplementary material for: Stable isotopes reveal patterns of diet and mobility in the last Neandertals and first modern humans in Europe
Source: Sci Rep. 2019 Mar 14;9:4433. doi: 10.1038/s41598-019-41033-3 (PMC6418202; doi:10.1038/s41598-019-41033-3)
Supplement: Supplementary file 1 — Supplementary Dataset [file 41598_2019_41033_MOESM1_ESM.pdf]

Stable isotopes reveal patterns of diet and mobility in the last Neandertals and first modern humans in Europe

SUPPLEMENTARY INFORMATION

Christoph Wißing, Hélène Rougier, Chris Baumann, Alexander Comeyne, Isabelle Crevecoeur, Dorothée G. Drucker, Sabine Gaudzinski-Windheuser, Mietje Germonpré, Asier Gómez-Olivencia, Johannes Krause, Tim Matthies, Yuichi I. Naito, Cosimo Posth, Patrick Semal, Martin Street, Hervé Bocherens

Table of Contents

Supplementary Data

Supplementary Data 1	The Troisième caverne of Goyet
Supplementary Data 2	The herbivore assemblage from the Troisième caverne of Goyet
Supplementary Data 3	Scladina
Supplementary Data 4	Spy
Supplementary Data 5	Lommersum
Supplementary Data 6	Chemical characteristics of the collagen
Supplementary Data 7	Protein reconstruction for the Goyet UPMHs
Supplementary Data 8	Statistical analysis of the horse and mammoth isospaces

Supplementary Tables

Supplementary Table S1	List of UPMHs and Neandertals providing detailed chemical information
Supplementary Table S2	List of stable isotopic data and related ^{14}C dates from Late Pleistocene herbivorous and carnivorous mammal species from the Troisième caverne of Goyet, Scladina and Spy
Supplementary Table S3	List of stable isotopic data from herbivorous and carnivorous mammal species from the site of Lommersum
Supplementary Table S4	Herbivore assemblages from the Troisième caverne of Goyet and Spy
Supplementary Table S5	Mammoth assemblage from the Troisième caverne of Goyet
Supplementary Table S6	Age distribution of the mammoth molars from the Troisième caverne of Goyet and Spy

Supplementary Figures

Supplementary Figure 1	Cluster analysis of Late Pleistocene herbivorous species from the Troisième caverne of Goyet, Scladina and Spy based on $\delta^{15}N$ and $\delta^{13}C$ bone collagen values
Supplementary Figure 2	Proportion vs. density plots showing the probability distributions of the amount of different protein sources (species) in the diet of the Belgian UPMHs
Supplementary Figure 3	Diagnostic matrix plots summarizing the statistical dependencies between different protein sources for the UPMHs from the Troisième caverne of Goyet
Supplementary Figure 4	Age groups of the mammoths from Goyet and Spy
Supplementary Figure 5	Box plots of the relative contributions of different prey species in the diet of the Saint-Césaire I Neandertal (France)
Supplementary Figure 6	Diagnostic matrix plots summarizing the statistical dependencies between different protein sources for the Saint-Césaire I Neandertal (France)
Supplementary Figure 7	Proportion vs. density plot showing the probability distributions of the amount of different protein sources (species) in the diet of the Saint-Césaire I Neandertal
Supplementary Figure 8	Box plots of the relative contribution of mammoth meat for different large predators
Supplementary Figure 9	Horse and mammoth carbon and nitrogen stable isotopic data presented in chronological order
Supplementary Figure 10	Upper first milk molar of a juvenile mammoth (2777-6) from the Troisième caverne of Goyet, Belgium (Dupont collection, Royal Belgian Institute of Natural Sciences). Photo credit: Royal Belgian Institute of Natural Sciences

Supplementary Data 1-The Troisième caverne of Goyet

The Goyet cave system is located within a limestone massif above the Samson River, a tributary of the Meuse River, in the Mozet Commune of the Walloon Region in Belgium. Goyet consists of several cave sites and the fossil remains used in this study all come from the Troisième caverne. Most of the cave sediments were excavated early on by Edouard Dupont in 1868[1]. Information about the stratigraphical origins of the material is evidently quite simplistic, having followed the state of the art at the time. Most of the archeological material collected by E. Dupont comes from three of the five "fauna-bearing levels" he identified. This archeological material was believed to belong to a single archeological cultural group called the "Goyet type", despite the fact that the artifacts came from multiple layers [1,2]. The presence of human remains was mentioned by Dupont (1872) but Neandertal remains were not identified until 2004[3-5]. Additionally, modern human remains from several periods of the Upper Palaeolithic were later identified[4,6]. All of the human and faunal material from the Dupont excavation is housed at the Royal Belgian Institute of Natural Sciences in Brussels. The ongoing dating program has confirmed observations made in previous studies[7-9] that each of the five "layers" described at the Troisième caverne contains material from different periods. However, the site has generated one of the richest archeological and anthropological records in NW Europe, with several Middle and Upper Palaeolithic techno-complexes (Mousterian, Lincombian-Ranisian-Jerzmanowician, Aurignacian, Gravettian, and Magdalenian), including a considerable faunal assemblage[2,10-14].

Supplementary Data 2-The herbivore assemblage from the Troisième caverne of Goyet

Material and methods

Dupont distinguished five "fauna-bearing levels" in the Troisième caverne of Goyet[1]. He recovered numerous Pleistocene mammal bones, human remains and large quantities of Middle and Upper Palaeolithic artifacts from these layers[5,6-10]. Many of the bones display cut marks, were broken for marrow extraction or carry traces of ochre[5,28]. The dispersion of the AMS dates and the refitting of the human bones originating from different levels point out the mixed content of the layers recognized by Dupont[5,7,8]. Here, the herbivore remains from the upper four fauna-bearing levels from the Troisième caverne of Goyet are treated as one assemblage, bearing in mind that each level is likely to have accumulated over an extended time span. The assemblage from the fifth fauna-bearing level contains remains from cave lion and cave bear[1].

The skeletal elements of the Goyet herbivore assemblage were counted in Number of Identified Specimens (NISP) and in Minimum Number of Individuals (MNI) based on Lyman[15]. For the palaeobiological aspects of the mammoth, we used data on ageing and reproduction in extant elephants. Modern day African and Asian elephants are alike in several aspects of their biology and behavior. They have similar gestation periods and birth rates and the maximum life span of both species is about 60 years[16-18]. It is probable that the habits and life cycle of the woolly mammoth were comparable, to a certain extent, to those of elephants today[16,17,19-21]. Based on detailed analyses of the microstructure of mammoth tusks, the gestation length of mammoth was estimated to have been 20-22 months[21,22], slightly shorter than in the extant African elephant, which usually has a gestation period of about 22 months[23]. In mammoths, conception is likely to have occurred in late spring and giving birth in early spring[22,24]. The study of Grigoriev et al.[22] furthermore suggests an

inter-birth interval of about four years. During this interval, the previously born calf would have been nursing. In mammoths, weaning probably occurred shortly before the next calf was born, just as in elephants[22]. Maschenko[20] and Rountrey[24] describe in detail the development of first milk molars from mammoth calves discovered at the Gravettian Kostenki-21 site (32572-8) and at the Yuribei River, Yamal Peninsula, Russia (Lyuba)[24]. The Kostenki-21 calf was between one and two months old when it died[20]; the Lyuba calf died at an age of 35 days[24]. Laws[25] established 30 age classes (I-XXX) for the African elephant, basing these upon the progress of eruption and wear of the jugal teeth and then allocating real ages to these groups. The mammoth molars were identified using the measurements and number of plates, following Musil and Germonpré[26,27]. To age the Goyet mammoths, Laws'[25] technique as modified by Craig in Haynes[17] was used. The mammoth molars were attributed an "African elephant year" (a.e.y.) age, using the data of Craig in Haynes[17] (Table A8).

Results

The herbivore assemblage from Goyet is dominated by horse and reindeer remains. The next two best represented species are woolly rhinoceros and mammoth, although the remains of these latter mammals occur in much lower frequencies (Table S4). Except for the mammoth, the herbivore remains derive mainly from adult individuals[10]. Mammoth ivory fragments and flakes, many displaying ochre traces and cut marks, constitute about 60% of the NISP of the mammoth assemblage from Goyet. In addition, several ivory beads and pendants have been described from Goyet, dating from the Aurignacian and Gravettian[29]. Apart from the ivory, the mammoth assemblage is dominated by remains from the skull (47.5% of the NISP, excluding ivory) followed by long bones fragments (42.5 % of the NISP, excluding ivory). Elements from the feet are missing (Table S5). Based on the dental and postcranial material in the mammoth assemblage, three age groups can be distinguished: juvenile, subadult and adult. The Goyet mammoth assemblage includes a complete upper first milk molar (2777-6)[10] with limited wear of the plates and cement fills in the interplate gaps (Supplementary Fig. 11). It is very similar in its development to the first milk molar from the Kostenki-21 mammoth calf (ZIN 32572-8) and the Lyuba mammoth calf. The Goyet calf probably died at a similar age to these two animals, when about one to two months old. At Goyet, the NISP of mammoth calves younger than four years old amounts to nine (MNI: 3) (Table S5, Table S6, Supplementary Fig. 5 and 11). The age profile of the Goyet mammoth assemblage is dominated by young nursing calves (< 4 a.e.y.), just as is the case for the Spy assemblage[30]. Older calves (5-12 a.e.y.) and subadults (13-24 a.e.y.) are better represented than adults (> 24 a.e.y.) (Table S4, Supplementary Fig. 5).

Discussion

As the Troisième caverne of Goyet was also used by cave hyenas as a den, it is likely that a certain amount of the herbivore body parts were transported to the den by this carnivore. Nevertheless, traces of human manipulation have been identified on the bones of horse, reindeer, mammoth and rhinoceros [7,10]. Furthermore, it has been argued that it is unlikely that cave hyenas would hunt mammoth calves and haul the heads uphill to their den[33]. Human hunting of elephants is in general characterized by a preference for calves[31,32], likely related to the fact that their young age makes hunting them easier. Human hunters also succeed better than carnivores at separating the calves from their protective mothers. Other advantages would have been the ease of carrying the carcasses of calves compared to the burden of transporting the bodies of adult mammoths to the camp site (maybe limited to carrying lumps of meat) and nutritional considerations (better taste, specific

nutrients, higher quality fat i.e. omega-3 fatty acids related to the intake of cow's milk)[31-36]. Hunting of adult mammoths, often cows, can be related to the need for skeletal parts like skulls, jaws and long bones for food (meat, fat), construction purposes or fuel[37-39], or for tusks as a raw material for tools and ornaments[40,41]. For these reasons, we expect skeletal bone remains from adult mammoths to be underrepresented in the zooarcheological record. In the Goyet assemblage, the calves younger than four a.e.y. were likely to still have been suckling at their age at death. It is likely that nursing mammoth calves remained very close to their mother and were also offered protection by the whole herd as are modern day elephant calves[23], thereby reducing the risk of predator-related mortality. At Goyet, the main target for the human hunters was likely to have been young nursing mammoth calves, maybe together with their mothers. One mammoth calf (2777-6) (Supplementary Fig. 11) died at one to two months in age, probably during the spring. At Goyet, just as at Spy[30,33], it appears that the heads of mammoths were preferentially brought to the cave and to a lesser extent long bones. The mammoth foot pads seem to have been abandoned or utilized elsewhere. At the Gravettian Krems-Wachtberg open-air site in Austria, the mammoth assemblage is also dominated by nursing mammoth calves[42]. In contrast to the cave assemblages from Goyet and Spy, the Krems-Wachtberg assemblage derives from a geologically short time period, with the mammoth assemblage likely to represent the killing of one mammoth family unit[42]. It must be pointed out that both the Goyet and Spy mammoth assemblages represent palimpsests and likely reflect several hunting episodes[33].

Conclusion

Combining all this evidence permits us to propose that the mammoth calves (and possibly their mothers) were killed and that their carcasses, heads or body parts were then transported to the Troisième caverne of Goyet by human hunters. The age at death of one calf implies that at least one hunt took place during the spring.

Supplementary Data 3-Scladina

The Scladina cave site is located in the Condroz region (commune of Andenne, province of Namur) south of the Sambre and Meuse valleys in Belgium. Scladina lies in a small valley adjacent to the Meuse River in the village of Sclayn. The site was discovered in 1971 and since 1978 has been the subject of ongoing scientific excavations[46,47]. Scladina is a major occupational site of the Middle Palaeolithic in NW Europe and was therefore classified as a "Site exceptionnel de Wallonie" in 1996. Besides an extensive Late Pleistocene record[14,48] associated with Middle Palaeolithic artifact assemblages[2,47], remains of a Neandertal child were discovered in complex 4A[49]. These remains are significantly older than the human remains considered in the present study and are dated to ca. 86-88,000 yrs BP[47]. In this study, mammoth steppe faunal remains come from complex 1A, which has an age of around 40,000-37,000 ka ^{14}C BP[14]. This faunal assemblage is associated with a Mousterian stone tool techno-complex[14]. Analysis of the carbon and nitrogen isotopic compositions of an assemblage representative of herbivorous and carnivorous species was already performed in 1997[50]. The species considered are the woolly mammoth (*Mammuthus primigenius*), woolly rhinoceros (*Coelodonta antiquitatis*), horse (*Equus ferus*), large bovids, i.e. aurochs (*Bos primigenius*) or steppe bison (*Bison priscus*), cave hyena (*Crocuta crocuta*), brown bear (*Ursus arctos*), and cave bear (*Ursus spelaeus*) (Table S1).

Supplementary Data 4-Spy

The site of Spy is one of the richest Palaeolithic sites in Belgium. The small cave is located in the Ardennes, close to the Meuse Valley (Fig. 1). The Spy cave lies on the left bank of the Orneau, a tributary of the Sambre. In 1885, two Neandertal skeletons were discovered by M. De Puydt and M. Lohest[51]. Almost all of the human and faunal material from their excavations is housed at the Royal Belgian Institute of Natural Sciences in Brussels. Unfortunately, most of the mammal material does not have a stratigraphic attribution[30,33]. The remains of Neandertal individuals Spy I and II were investigated for their carbon and nitrogen stable isotopic composition[52]. The $\delta^{13}C$ and $\delta^{15}N$ isotopic values of the Spy VI child, discovered several years ago during the sorting of the faunal collections[53], are presented here. The Spy Neandertals are among the youngest directly dated Neandertals discovered to date[51,54,55]. The four best represented species in the Spy herbivore assemblage (combining material with and without stratigraphic provenance) are the same ones as in the Goyet assemblage: horse, mammoth, rhinoceros, and reindeer[30,33] (Table S2). The age distribution of the mammoth molars from Spy is similar to the distribution of those from the Troisième caverne of Goyet and is also dominated by calves that were still nursing when killed (Table S4, Supplementary Fig. 6). The Spy rhinoceros assemblage consists mainly of young adults and nursing calves. Prehistoric people manipulated some rhinoceros material as shown by ochre traces and a cut marked rhinoceros humerus[30].

Supplementary Data 5-Lommersum

The open-air site of Lommersum is situated in a small valley in the foothills of the Eifel around 40 km southwest of Cologne, Nordrhein-Westfalen province (Fig. 1). The site is one of very few open-air Aurignacian sites in Germany[43,44]. For the Northern parts of Central Europe, there are currently almost no sites with adequate quantities of well-preserved faunal remains dating to the early UP. Lommersum was discovered in 1969 and excavations by Joachim Hahn took place from 1971 to 1974 as well as in 1977 and 1978. The results are published in a monograph[43]. The archeological horizon IIc is the most important one as it contains the highest artifact density as well as structures such as a hearth[43]. Among the faunal remains, the most abundant species are the reindeer (*Rangifer tarandus*) and horse (*Equus* sp.) but carnivores such as the cave lion (*Panthera spelaea*), wolf (*Canis lupus*), wolverine (*Gulo Gulo*), and fox (*Vulpes lagopus*) are also represented. Mammoth (*Mammuthus primigenius*) is only represented as worked ivory[43]. The latest AMS radiocarbon dates indicate an age between 32,250 ± 500 and 35,100 ± 650 ^{14}C yr BP[45]. The conventional ^{14}C dates from Lommersum initiated by Hahn span from 29,200 ± 850 up to 33,420 ± 500 ^{14}C yr BP. Hahn discussed the age of layer IIc between 29,000 and 32,000 ^{14}C yr BP[43]. All samples came from layer IIc except one horse (*Equus* sp.) (Lom-22) from complex IIb.

Supplementary Data 6-Chemical characteristics of the collagen

For all of the Goyet samples analyzed, the collagen preservation fulfilled the conditions for reliable biogenic stable carbon and nitrogen isotopic values. The detailed chemical characteristics are summarized in Table S1. The carbon content in collagen ranges between 29.5 % (Q48-1) and 45.6 % (Q116-1). The nitrogen content in collagen ranges from 10.1 % (Q48-1) to 16.3 % (Q116-1). The C:N

ratio in collagen is between 3.3 and 3.4. The yield is always higher than 1 %. Based on comparisons with collagen extracted from fresh bones, the accepted maximum percentage of sulphur in collagen is 0.26 %. In the present study, this prerequisite was not fulfilled for all of the Neandertal specimens, and therefore the sulphur stable isotopic values of the following individuals were not taken into account: Q48-1, Q119-2, Q305-7, Q376-9 and Q376-25.

Isotopic results

$\delta^{15}N$ and $\delta^{13}C$

We analyzed the $\delta^{15}N$ and $\delta^{13}C$ stable isotope composition of two Upper Palaeolithic modern human (UPMH) individuals (Q116-1 and Q376-3). The respective $\delta^{13}C$ values of -19.1 ‰ and -18.8 ‰ were obtained for individuals Q116-1 and Q376-3. The $\delta^{15}N$ values were 10.9 ‰ for Q116-1 and 11.4 ‰ for Q376-3 (Table 1). The six Goyet Neandertal specimens analyzed for their $\delta^{15}N$ and $\delta^{13}C$ stable isotope composition yielded $\delta^{13}C$ values between -19.0 ‰ and -19.7 ‰ (mean -19.3 ‰) and $\delta^{15}N$ values between 11.3 ‰ and 12.5 ‰ (mean 11.8 ‰). The Spy 646a (Spy VI) individual yielded a $\delta^{13}C$ value of -19.8 ‰ and a $\delta^{15}N$ value of 12.5 ‰ (Table 1).

$\delta^{34}S$

The $\delta^{34}S$ values for the analyzed Goyet UPMHs were 8.6 ‰ (Q116-1) and 4.4 ‰ (Q376-3). The values for the Goyet Neandertals (n=11) span from 7.5 ‰ (Q305-4) to 11.6 ‰ (Q376-20) with a mean of 10.2 ‰. The $\delta^{34}S$ values for the Spy Neandertals are 3.6 ‰ for Spy 94a and 2.6 ‰ for Spy 646a (Spy VI). The faunal $\delta^{34}S$ values from Goyet (n=27) span between -7.2 and 8.4 ‰ (mean 1.2 ‰, s.d. 4.1 ‰). The faunal $\delta^{34}S$ values from Scladina (n=23) range from -17.0 ‰ to 11.8 ‰ (mean 2.4 ‰, s.d. 5.8 ‰). In addition, two faunal $\delta^{34}S$ values from Spy are provided here with values of 5.5 ‰ (specimen IV2A 4207) and 2.5 ‰ (specimen Spy D4 19B 121 1480) (Table S2).

Lommersum

$\delta^{15}N$ and $\delta^{13}C$

The $\delta^{13}C$ values range between -20.9 ‰ for a horse (Lom-20) to -18.3 ‰ for a reindeer (Lom-8) (mean -19.74 ‰, s.d. 0.9 ‰). The $\delta^{15}N$ values range from 2.0 ‰ for a reindeer (Lom-7) to 8.5 ‰ for the cave lion (Lom-15) (mean 4.8 ‰, s.d. 2.0 ‰) (Table S2).

$\delta^{34}S$

Seven samples provided collagen that fulfilled the criteria for $\delta^{34}S$ values. Four samples contained a higher percentage of sulphur in collagen (> 0.26 %) and were therefore excluded from further interpretation. The maximum $\delta^{34}S$ value was obtained on the reindeer Lom-4 (4.7 ‰) while the lowest value was from Lom-2 (1.4 ‰). The average $\delta^{34}S$ is 3.4 ‰ (s.d. 1.5 ‰) (Table S2).

Supplementary Data 7-Protein reconstruction for the Goyet UPMHs

In Supplementary Fig. 4 we present the proportion vs. density plots for each potential prey of the UPMHs from Goyet. On the x-axis, the proportion is given and on the y-axis, the density/probability is provided. For example, the density for cave bear is the highest at the lowest proportion, and decreases exponentially; therefore, a low amount of cave bear as potential dietary protein has a much higher probability than a higher amount. The same is true for the horses and the bovines,

although in a less extreme way than for the cave bear. The rhinoceros (for individual Q116-1), mammoth and reindeer have a bell shaped distribution of proportion vs. density. This implies that the highest probability for a specific dietary amount is at a given value (i.e. at the bell's peak) and a higher or a lower proportion is less probable. To gain a more comprehensive understanding of potential statistical dependencies between each prey species, we generated a diagnostic matrix plot for each individual (Supplementary Fig. 5). The matrix plots show the correlations, positive or negative, between the different dietary protein sources. In general, the higher the correlations, the greater the total probabilities range for a potential prey species. For both individuals (Q116-1 and Q376-3), the three highest correlations are negative ones, between mammoth and rhinoceros, between rhinoceros and reindeer, and between bovines and reindeer. The negative correlation between mammoth and rhinoceros implies that a higher relative amount of mammoth would signify a lower amount of rhinoceros, and alternatively an increasing amount of rhinoceros would mean a lower quantity of mammoth. If we additionally consider the negative correlation between rhinoceros and reindeer, we see that a lower amount of rhinoceros implies a higher amount of reindeer and mammoth, since negative correlations exist between rhinoceros and mammoth and between rhinoceros and reindeer. Finally, it is hard to narrow down the probability range for these species due to the above-mentioned correlations. However, the general trends are evident, so that the most and least important prey species can be sufficiently identified through this approach.

Supplementary Data 8-Statistical analysis of the horse and mammoth isospaces

We ran statistical analyses for the Belgian and Swabian Jura samples. The sample sizes from Ziegeleigrube Coenen and Lommersum were too unequal because at both sites, mammoth is underrepresented.

Belgium:
Carbon and nitrogen isotopes show no difference between the variances of horse and mammoth (δ^{13}C: Levene's test, F = 3.63, df = 1.17, P = 0.0736; δ^{15}N: Levene's test, F = 0.65, df = 1.17, P = 0.4306). The sample sizes were equal, which allowed us to run a t-test (with JMP 14). Carbon values do not differ between the two species (t-test, t = 0.41, df = 17, P = 0.688), while nitrogen values differ significantly (t-test, t = -5.10, df = 17, P < 0.001).

Swabian Jura:
Carbon and nitrogen isotopes show no difference between the variances of the two species (δ^{13}C: Levene's test, F = 0.92, df = 1.46, P = 0.343; δ^{15}N: Levene's test, F = 2.67, df = 1.46, P = 0.1089). The sample sizes were equal, which allowed us to run a t-test (with JMP 14). For both isotopic variables, significant differences are present (δ^{13}C: t-test, t = 3.97, df = 46, P = 0.0002; δ^{15}N: t-test, t = -6.78, df = 46, P < 0.001).

However, the niches of the two species seem to become less distinct during the early UP. To demonstrate this, we calculated the distances between the means of both species in both regions. The highest dynamics are seen in the nitrogen isotopic values. The Belgian horses and mammoths show a distance of 2.81 ‰ in their mean values, while the Swabian Jura species show a difference of only 2.06 ‰. The distance between the mean values of both niches is lower in the younger sites of the Swabian Jura. This indicates that the niches are closer to each other and not as distinct as at the older Belgian sites.

To demonstrate overlapping of the mammoth and horse niches, we calculated the distances beteween the mammoth's minimum values and the horse's maximum values. The Belgian species show an overlap (distance between $Mammoth_{min}$ and $Horse_{max}$) of 0.34 ‰, while the Swabian Jura species show a more important overlap of 0.94 ‰.

Distance between niches

		$\delta^{13}C$ [‰]	$\delta^{15}N$[‰]
$Mammoth_{mean}$	Belgium	-21.26	8.45
$Horse_{mean}$		-21.18	5.64
	**distance**	**-0.09**	**2.81**
$Mammoth_{mean}$	Swabian Jura	-21.15	8.74
$Horse_{mean}$		-20.74	6.69
	**distance**	**-0.41**	**2.06**

Overlap of niches

		$\delta^{15}N$ [‰]
$Horse_{max}$	Belgium	7.00
$Mammoth_{min}$		6.66
	**overlap**	**0.34**
$Horse_{max}$	Swabian Jura	8.92
$Mammoth_{min}$		7.98
	**overlap**	**0.94**

Literature

1 Dupont, E. L'homme pendant les âges de la pierre dans les environs de Dinant-sur-Meuse. *C. Muquardt Ed., Bruxelles* (1872).

2 Di Modica, K., Toussaint, M., Abrams, G. & Pirson, S. The Middle Palaeolithic from Belgium: Chronostratigraphy, territorial management and culture on a mosaic of contrasting environments. *Quaternary International* **411**, 77-106 (2016).

3 Rougier, H. *et al.* New data from an old site: Neandertals at Goyet (Belgium) and their mortuary behavior. *American Journal of Physical Anthropology* **147**, 252-253 (2012).

4 Rougier, H. *et al.* The First Upper Paleolithic Human Remains from Belgium: Aurignacian, Gravettian and Magdalenian Fossils at the "Troisième caverne" of Goyet. *PaleoAnthropology* **2013**, A 33 (2013).

5 Rougier, H. *et al.* Neandertal cannibalism and Neandertal bones used as tools in Northern Europe. *Scientific Reports* **6**, 29005 (2016).

6 Posth, C. *et al.* Pleistocene Mitochondrial Genomes Suggest a Single Major Dispersal of Non-Africans and a Late Glacial Population Turnover in Europe. *Current Biology : CB* **26**, 827-833 (2016).

7 Germonpré, M. The Magdalenian upper horizon of Goyet and the late Upper Palaeolithic recolonisation of the Belgian Ardennes. *Bulletin de l'Institut royal des Sciences naturelles de Belgique, Sciences de la Terre* **67**, 167-182 (1997).
8 Germonpré, M. & Sablin, M. The cave bear (*Ursus spelaeus*) from Goyet, Belgium. The bear den in Chamber B (bone horizon 4). *Bulletin de l'Institut royal des Sciences naturelles de Belgique, Sciences de la Terre* **71**, 209-233 (2001).
9 Stevens, R. E., Germonpré, M., Petrie, C. A. & O'Connell, T. C. Palaeoenvironmental and chronological investigations of the Magdalenian sites of Goyet Cave and Trou de Chaleux (Belgium), via stable isotope and radiocarbon analyses of horse skeletal remains. *Journal of Archaeological Science* **36**, 653-662 (2009).
10 Comeyne, A. Taphonomy, osteometry and archaeozoology of the Pleistocene herbivores from the third horizon of the Goyet cave, Belgium. *Master Thesis, Universiteit Gent* (2013).
11 Flas, D. The Middle to Upper Paleolithic transition in Northern Europe: the Lincombian-Ranisian-Jerzmanowician and the issue of acculturation of the last Neanderthals. *World Archaeology* **43**, 605-627 (2011).
12 Flas, D. The Chronocultural Sequence of Belgian Complexes in the European Aurignacian Context. *Aurignacian Genius: Art, Technology and Society of the First Modern Humans in Europe, Proceedings of the International Symposium, April 08-10 2013, New York University*, 57-75 (2015).
13 Otte, M. & Noiret, P. Le Gravettien du Nord-Ouest de l'Europe. *Paleo* **19**, 243-256 (2007).
14 Pirson, S. *et al.* Chronostratigraphic context of the Middle to Upper Palaeolithic transition: Recent data from Belgium. *Quaternary International* **259**, 78-94 (2012).
15 Lyman, R. L. Vertebrate taphonomy. Cambridge Manuals in Archaeology. *Cambridge University Press*, *Cambridge* (1994).
16 Haynes, G. Finding meaning in mammoth age profiles. *Quaternary International* **443**, 65-78 (2017).
17 Haynes, G. Mammoths, Mastodonts, and Elephants. Biology, Behavior, and the Fossil Record. *Cambridge University Press, Cambridge* (1991).
18 Olivier, R. C. D. Ecology and behavior of living elephants: bases for assumptions concerning the extinct woolly mammoth. *Hopkins, D.M., Matthews, J.V., Schweger, C.E. & Young, S.B. (Eds.), Paleoecology of Beringia. Academic Press, New York* 291-305(1982).
19 Saunders, J. J. A model for man–mammoth relationships in Late Pleistocene North America. *Canadian Journal of Anthropology* **1**, 87-98 (1980).
20 Maschenko, E. N. Individual development, biology and evolution of the woolly mammoth. *Cranium* **19**, 1-120 (2002).
21 Fisher, D. C. *et al.* X-ray computed tomography of two mammoth calf mummies. *Journal of Paleontology* **88**, 664-675 (2015).
22 Grigoriev, S. E. *et al.* A woolly mammoth (*Mammuthus primigenius*) carcass from Maly Lyakhovsky Island (New Siberian Islands, Russian Federation). *Quaternary International* **445**,89-103 (2017).
23 Moss, C. Elephant Memories: Thirteen Years in the Life of an Elephant Family. *William Morrow, New York* (1988).
24 Rountrey, A. N. *et al.* Early tooth development, gestation, and season of birth in mammoths. *Quaternary International* **255**, 196-205 (2012).
25 Laws, R. M. Elephants and their Habitats. *Clarendon Press, Oxford* (1975).

26 Germonpé, M. Taphonomy of Pleistocene mammal assemblages of Flemish Valley, Belgium. *Bulletin de l'Institut royal des Sciences naturelles de Belgique, Sciences de la Terre* **63**, 271-309 (1993).
27 Musil, R. Die Mammutmolaren von Předmostí (ČSSR). *Paläontologische Abhandlungen* **3**, 1-192 (1968).
28 Germonpré, M. & Hämäläinen, R. Fossil bear bones in the Belgian Upper Palaeolithic: the possibility of a proto-bear ceremonialism. *Arctic Anthropology* **44**, 1-30 (2007).
29 Otte, M. Le paléolithique supérieur ancien en Belgique. *Monographies d'Archéologie Nationale* **5**, 1-684 (1979).
30 Germonpré, M., Udrescu, M. & Fiers, E. The fossil mammals of Spy. *Rougier, H. & Semal, P. (Eds.), Spy cave. 125 years of multidisciplinary research at the Betche aux Rotches (Jemeppe-sur-Sambre, Province of Namur, Belgium), Volume 1. Anthropologica et Praehistorica* **123/2012**, *Royal Belgian Institute of Natural Sciences, Royal Belgian Society of Anthropology and Praehistory & NESPOS Society, Brussels,* 298-327 (2013).
31 Reshef, H. & Barkai, R. A taste of an elephant: The probable role of elephant meat in Paleolithic diet preferences. *Quaternary International* **379**, 28-34 (2015).
32 Agam, A. & Barkai, R. Elephant and Mammoth Hunting during the Paleolithic: A Review of the Relevant Archaeological, Ethnographic and Ethno-Historical Records. *Quaternary* **1**, 3 (2018).
33 Germonpré, M., Udrescu, M. & Fiers, E. Possible evidence of mammoth hunting at the Neanderthal site of Spy (Belgium). *Quaternary International* **337**, 28-42 (2014).
34 Guil-Guerrero, J. L. *et al.* The Fat from Frozen Mammals Reveals Sources of Essential Fatty Acids Suitable for Palaeolithic and Neolithic Humans. *PLoS ONE* **9, e84480** (2014).
35 Guil-Guerrero, J. L. Evidence for chronic omega-3 fatty acids and ascorbic acid deficiency in Palaeolithic hominins in Europe at the emergence of cannibalism. *Quaternary Science Reviews* **157**, 176-187 (2017).
36 Agam, A. & Barkai, R. Not the brain alone: The nutritional potential of elephant heads in Paleolithic sites. *Quaternary International* **406**, 218-226 (2016).
37 Germonpré, M., Sablin, M., Khlopachev, G. A. & Grigorieva, G. V. Possible evidence of mammoth hunting during the Epigravettian at Yudinovo, Russian Plain. *Journal of Anthropological Archaeology* **27**, 475-492 (2008).
38 Beresford-Jones, D. G. *et al.* Burning wood or burning bone? A reconsideration of flotation evidence from Upper Palaeolithic (Gravettian) sites in the Moravian Corridor. *Journal of Archaeological Science* **37**, 2799-2811 (2010).
39 Péan, S. Mammouth et comportements de subsistance à l'Épigravettien : analyse archéozoologique du secteur de la fosse no 7 associée à l'habitation no 1 de Mezhyrich (Ukraine). *L'Anthropologie* **119**, 417-463 (2015).
40 Münzel, S. C., Wolf, S., Drucker, D. G. & Conard, N. J. The exploitation of mammoth in the Swabian Jura (SW-Germany) during the Aurignacian and Gravettian period. *Quaternary International* **445**, 184-199 (2017).
41 Wolf, S. & Heckel, C. Ivory Ornaments of the Aurignacian in Western Europe: Case studies from France and Germany. *L'Anthropologie* **122,** 348-373 (2018).
42 Fladerer, F. A. A calf-dominated mammoth age profile from the 27 kyBP stadial Krems-Wachtberg site in the middle Danube valley. *Reumer, J.W.F., De Vos, J. & Mol, D. (Eds.), Advances in Mammoth Research (Proceedings of the Second International Mammoth Conference, Rotterdam, May 16-20 1999), DEINSEA* **9**, 135-158 (2003).

43 Hahn, J. Genese und Funktion einer jungpaläolithischen Freilandstation Lommersum im Rheinland. *Rheinische Ausgrabungen* **29** (1989).

44 Pastoors, A. & Tafelmaier, Y. What about flakes? Flake production and core reduction strategies in the Aurignacian of the Rhineland: Lommersum IIc (North Rhine Westphalia) and Wildscheuer III (Hessen). *Pastoors, A. & Peresani, M. (Hg.), Flakes not Blades* (2012).

45 Matthies, T. Subsistence strategies during the Early Upper Palaeolithic of northern Central Europe: A re-analysis of the faunal remains from Lommersum (Germany). *Proceedings of the European Society for the study of Human Evolution* **1**, 122 (2012).

46 Abrams, G. *et al.* Les os brûlés de l'ensemble sédimentaire 1A de Scladina (Andenne, Belgique) : apports naturels ou restes de foyer(s) néandertalien(s) ? *Notae Praehistoricae* **30**, 5-13 (2010).

47 Bonjean, D. *et al.* A new Cambrian black pigment used during the late Middle Palaeolithic discovered at Scladina Cave (Andenne, Belgium). *Journal of Archaeological Science* **55**, 253-265 (2015).

48 Pirson, S. *et al.* Belgian cave entrance and rock-shelter sequences as palaeoenvironmental data recorders: The example of Walou Cave. *Geologica Belgica* **3-4**, 275-286 (2006).

49 Toussaint, M., Otte, M., Bonjean, D., Falguères, C. & Yokoyama, Y. Les restes humains néandertaliens de la couche 4A de la grotte Scladina (Andenne, Belgique). *Comptes-Rendus de l'Académie des Sciences de Paris* **326**, 737-742 (1998).

50 Bocherens, H. *et al.* Paleobiological Implications of the Isotopic Signatures (13C, 15N) of Fossil Mammal Collagen in Scladina Cave (Sclayn, Belgium). *Quaternary Research* **48**, 370-380 (1997).

51 Semal, P. *et al.* New data on the late Neandertals: direct dating of the Belgian Spy fossils. *American Journal of Physical Anthropology* **138**, 421-428 (2009).

52 Bocherens, H., Germonpré, M., Toussaint, M. & Semal, P. Stable isotopes. *Rougier, H. & Semal, P. (Eds.), Spy cave. 125 years of multidisciplinary research at the Betche aux Rotches (Jemeppe-sur-Sambre, Province of Namur, Belgium), Volume 1. Anthropologia et Praehistorica* **123/2012**, *Royal Belgian Institute of Natural Sciences, Royal Belgian Society of Anthropology and Praehistory & NESPOS Society, Brussels*, 357-370 (2013).

53 Crevecoeur, I. *et al.* The Spy VI child: a newly discovered Neandertal infant. *Journal of Human Evolution* **59**, 641-656 (2010).

54 Higham, T. *et al.* The timing and spatiotemporal patterning of Neanderthal disappearance. *Nature* **512**, 306-309 (2014).

55 Semal, P. *et al.* Radiocarbon dating of human remains and associated archaeological material. *Rougier, H. & Semal, P. (Eds.), Spy cave. 125 years of multidisciplinary research at the Betche aux Rotches (Jemeppe-sur-Sambre, Province of Namur, Belgium), Volume 1. Anthropologica et Præhistorica* **123/2012**, *Royal Belgian Institute of Natural Sciences, Royal Belgian Society of Anthropology and Praehistory & NESPOS Society, Brussels*, 331-356 (2013).

56 Bocherens, H. *et al.* Isotopic evidence for dietary ecology of cave lion (*Panthera spelaea*) in North-Western Europe: Prey choice, competition and implications for extinction. *Quaternary International* **245**, 249-261 (2011).

57 Wißing, C. *et al.* Isotopic evidence for dietary ecology of late Neandertals in North-Western Europe. *Quaternary International* **411**, 327-345 (2016).

58 Parnell, A. C., Inger, R., Bearhop, S. & Jackson, A. L. Source partitioning using stable isotopes: coping with too much variation. *PLoS ONE* **5**, e9672 (2010).

59 The-R-CoreTeam. R: A language and environment for statistical computing (2013).

60 Wißing, C., Matzerath, S., Turner, E. & Bocherens, H. Paleoecological and climatic implications of stable isotope results from late Pleistocene bone collagen, Ziegeleigrube Coenen, Germany. *Quaternary Research* **84**, 96-105 (2015).

Supplementary Table S1-List of UPMHs and Neandertals providing detailed chemical information

ID	Species	Description	%C	%N	C:N	Collagen yield (mg/g)	%S	C:S	N:S	Reference for $\delta^{13}C$ and $\delta^{15}N$
**Site: Goyet**										
Q116-1	*Homo sapiens*	Lt humerus diaphysis frag.	45.6	16.3	3.3	134.14	0.14	867	266	*
Q376-3	*Homo sapiens*	Rt humerus diaphysis frag.	43.3	15.4	3.3	117.8	0.14	814	248	*
C5-1	*Homo neanderthalensis*	Lt Parietal frag.	43.0	14.7	3.4	162.8	0.24	469	138	*
Q48-1	*Homo neanderthalensis*	Lt pubis frag.	29.5	10.1	3.4	90.6	2.1	37	11	*
Q53-4	*Homo neanderthalensis*	Rt humerus diaphysis frag. (humerus III)	42.9	15.1	3.3	150.9	0.19	603	182	57
Q55-1	*Homo neanderthalensis*	Lt clavicle frag.	36.9	12.9	3.3	137.0	0.19	522	157	57
Q55-4	*Homo neanderthalensis*	Rt tibia diaphysis frag. (tibia IV)	39.6	14.0	3.3	137.8	0.24	443	134	57
Q56-1	*Homo neanderthalensis*	Rt femur diaphysis frag. (femur I)	45.4	15.5	3.4		0.15	807	236	57
Q57-1	*Homo neanderthalensis*	Lt tibia diaphysis frag. (tibia II)	46.0	16.8	3.2		0.2	767	240	57
Q57-2	*Homo neanderthalensis*	Rt femur diaphysis frag. (femur II)	42.7	15.0	3.3	156.0	0.23	496	149	57
Q57-3	*Homo neanderthalensis*	Rt tibia diaphysis frag. (tibia VI)	43.8	15.4	3.3	159.5	0.17	693	209	57
Q 119-2	*Homo neanderthalensis*	Lt rib 7? shaft	38.9	13.8	3.3	130.0	0.45	228	69	*
Q305-4	*Homo neanderthalensis*	Lt tibia diaphysis frag. (tibia I)	47.1	16.7	3.3		0.16	785	239	57
Q305-7	*Homo neanderthalensis*	Rt tibia diaphysis frag. (tibia III)	41.9	14.9	3.3	101.8	0.29	381	116	57

ID	Species	Description	%C	%N	C:N	Collagen yield (mg/g)	%S	C:S	N:S	Reference for $\delta^{13}C$ and $\delta^{15}N$
Q374a-1	*Homo neanderthalensis*	Rt tibia diaphysis frag. (tibia V)	43.1	15.2	3.3	134.9	0.18	647	196	57
Q376-1	*Homo neanderthalensis*	Proximal manual. phalanx 2-4	46.7	17.0	3.2					57
Q376-20	*Homo neanderthalensis*	Rt humerus diaphysis frag. (humerus II)	39.8	14.0	3.3	133.7	0.25	429	130	57
Q376-9	*Homo neanderthalensis*	Rt rib 11	37.0	13.1	3.3	137.6	0.85	116	35	*
Q376-25	*Homo neanderthalensis*	Rt rib 11?	39.1	13.9	3.3	134.8	0.33	321	97	*
2878-2D	*Homo neanderthalensis*	Lower lt P4 (mandible 2878-8)	41.4	14.4	3.4	87.8				*
**Site: Spy**										
Spy 94a (Spy II?)	*Homo neanderthalensis*	Rt maxilla frag. attached to M3	43.7	15.2	3.4	77.5	0.16	728	217	52
Spy 430a (Spy II?)	*Homo neanderthalensis*	Rt middle 3rd manual phalanx	46.8	15.8	3.5	73.5				52
Spy 92b (Spy I?)	*Homo neanderthalensis*	Upper lt I1	47.1	16.7	3.3	90.3				52
Spy 572a (Spy I/II?)	*Homo neanderthalensis*	Rt scapula frag.	41.5	14.4	3.4					52
Spy 646a (Spy VI)	*Homo neanderthalensis*	Right hemi-mandible	43.1	15.4	3.3	47.4	0.17	676	207	*

Abbreviations: * = this study; Rt: right; Lt: left; frag.: fragment.
$\delta^{34}S$ values are all from this study; $\delta^{34}S$ values in red are not considered due to preservation issues. When collagen yield is not provided, collagen was extracted elsewhere but analysed by us.

Supplementary Table S2-List of stable isotopic data and related ^{14}C dates from Late Pleistocene herbivorous and carnivorous mammal species from the Troisième caverne of Goyet, Scladina and Spy

ID	Site	Species	Description	%C	%N	C/N	%S	C:S	N:S	$\delta^{34}S$	$\delta^{13}C$	$\delta^{15}N$	^{14}C Age (B.P.)	Ref. for dates	Ref. for $\delta^{13}C$ and $\delta^{15}N$
Goyet-A2-7	Goyet	*Panthera spelaea*	Phalanx	41.3	15.3	3.1					-18.5	8.4			56
Goyet-A3-1	Goyet	*Panthera spelaea*	Astragalus	44.7	15.5	3.4	0.19	626	185	-2.1	-18.7	8.4			56
Goyet-B5-1	Goyet	*Panthera spelaea*	Rt humerus	38.2	13.6	3.3	0.16	629	192	0.6	-18.7	7.3			56
Goyet-B5-2	Goyet	*Panthera spelaea*	Rt humerus	34.8	11.7	3.5					-20.7	9.6			56
Goyet-B5-4	Goyet	*Panthera spelaea*	Rt humerus	28.2	10.0	3.3					-19.5	6.3			56
Goyet-A1-6	Goyet	*Crocuta crocuta spelaea*	2nd metacarpal	41.4	15.2	3.2	0.17	649	204	1.4	-19.7	9.5			56
Goyet-A3-5	Goyet	*Crocuta crocuta spelaea*	3rd metatarsal	44.3	15.5	3.3	0.18	644	192	1.4	-19.2	9.1			56
Goyet-A3-6	Goyet	*Crocuta crocuta spelaea*	3rd metatarsal	43.2	15.5	3.2	0.19	612	189	4.5	-19.6	9.4			56
Goyet-A3-7	Goyet	*Crocuta crocuta spelaea*	3rd metatarsal	43.8	16.0	3.2	0.17	679	213	2.6	-19.6	9.8			56
Goyet-A3-8	Goyet	*Crocuta crocuta spelaea*	3rd metatarsal	43.5	15.8	3.2	0.17	696	216	4.3	-19.4	9.0			56
Goyet-B4-1	Goyet	*Crocuta crocuta spelaea*	Humerus	41.9	15.0	3.3					-19.8	7.7			56
SC1800	Scladina	*Crocuta crocuta spelaea*	Phalanx I	42.9	15.6	3.2	0.18	636	198	4.3	-20.2	8.8			50

ID	Site	Species	Description	%C	%N	C/N	%S	C:S	N:S	$\delta^{34}S$	$\delta^{13}C$	$\delta^{15}N$	^{14}C Age (B.P.)	Ref. for dates	Ref. for $\delta^{13}C$ and $\delta^{15}N$
SC1900	Scladina	*Crocuta crocuta spelaea*	Phalanx I	42.6	15.6	3.2	0.16	710	223	1.1	-19.6	10.1			50
SC2000	Scladina	*Crocuta crocuta spelaea*	Phalanx I	42.8	15.7	3.2	0.16	713	224	0.1	-19.7	8.2			50
SC2100	Scladina	*Crocuta crocuta spelaea*	Phalanx I	42.9	15.6	3.2					-19.4	9.5			50
SC1700	Scladina	*Crocuta crocuta spelaea*	Mandible	39.9	14.6	3.2					-19.8	9.6			50
SC2200	Scladina	*Crocuta crocuta spelaea*	Maxilla	42.0	15.4	3.2					-19.3	9.4			50
IV2A 13534	Spy	*Crocuta crocuta spelaea*	First molar	42.0	14.2	3.4					-19.9	11.90	42750 +850/-650 (GrA-44547)	55	57
Goyet-A3-3	Goyet	*Canis lupus*	4th metatarsal	43.5	15.6	3.3	0.18	644	198	1.7	-19.1	6.6			56
Goyet-A3-4	Goyet	*Canis lupus*	4th metatarsal	43.5	15.3	3.3	0.17	691	208	2.9	-18.8	7.2			56
SC30300	Scladina	*Canis lupus*	Lt pisiform	42.5	14.4	3.4	0.16	709	206	11.8	-20.4	6.6			56
Goyet-A2-3	Goyet	*Ursus spelaeus*	3rd metacarpal	39.9	14.3	3.3					-22.1	4.5			56
Goyet-A2-4	Goyet	*Ursus spelaeus*	3rd metacarpal	44.3	15.3	3.4					-21.7	3.4			56
Goyet-A2-5	Goyet	*Ursus spelaeus*	3rd metacarpal	39.2	13.5	3.4					-21.6	3.1			56
Goyet-A2-6	Goyet	*Ursus spelaeus*	3rd metacarpal	38.7	13.3	3.4					-22.1	3.0			56
Goyet-A3-20	Goyet	*Ursus spelaeus*	3rd metacarpal	43.5	16.0	3.2	0.15	749	236	-5.0	-21.8	2.8			56
Goyet-A3-22	Goyet	*Ursus spelaeus*	3rd metacarpal	40.7	14.6	3.2	0.16	663	204	-6.6	-21.5	2.6			56

ID	Site	Species	Description	%C	%N	C/N	%S	C:S	N:S	δ^{34}S	δ^{13}C	δ^{15}N	^{14}C Age (B.P.)	Ref. for dates	Ref. for δ^{13}C and δ^{15}N
Goyet-A3-23	Goyet	*Ursus spelaeus*	3rd metacarpal	39.4	13.7	3.4	0.16	651	194	-1.5	-21.9	4.3			56
Goyet-A3-24	Goyet	*Ursus spelaeus*	3rd metacarpal	40.1	14.4	3.2	0.14	759	234	-1.7	-21.5	4.9			56
Goyet-A3-25	Goyet	*Ursus spelaeus*	3rd metacarpal	41.2	15.7	3.1					-21.4	3.1			56
Goyet-A3-26	Goyet	*Ursus spelaeus*	3rd metacarpal	38.8	13.4	3.4					-21.6	5.8			56
Goyet-A3-27	Goyet	*Ursus spelaeus*	3rd metacarpal	43.5	15.5	3.3					-21.5	4.6			56
Goyet-A3-28	Goyet	*Ursus spelaeus*	3rd metacarpal	36.8	13.4	3.2					-22.3	5.3			56
Goyet-B4-9	Goyet	*Ursus spelaeus*	3rd metacarpal	41.2	14.9	3.2					-21.3	3.5			56
Goyet-B4-10	Goyet	*Ursus spelaeus*	3rd metacarpal	37.9	13.7	3.2					-21.8	4.4			56
Goyet-B4-11	Goyet	*Ursus spelaeus*	3rd metacarpal	39.3	13.3	3.4					-20.9	4.4			56
Goyet-B4-12	Goyet	*Ursus spelaeus*	3rd metacarpal	43.7	15.8	3.2					-20.9	3.7			56
Goyet-B4-13	Goyet	*Ursus spelaeus*	3rd metacarpal	42.2	15.3	3.2					-21.0	4.8			56
Goyet-B4-14	Goyet	*Ursus spelaeus*	3rd metacarpal	42.0	14.9	3.3					-21.8	4.5			56
Goyet-B4-15	Goyet	*Ursus spelaeus*	3rd metacarpal	43.0	15.3	3.3					-21.0	4.6			56
Goyet-B4-16	Goyet	*Ursus spelaeus*	3rd metacarpal	36.4	14.5	2.9					-21.0	6.0			56
Goyet-B4-17	Goyet	*Ursus spelaeus*	3rd metacarpal	41.8	14.5	3.4					-20.8	3.9			56
Goyet-B4-32	Goyet	*Ursus spelaeus*	Radius	40.7	14.8	3.2					-21.7	5.2			56
Goyet-B4-34	Goyet	*Ursus spelaeus*	3rd metacarpal	41.2	14.1	3.4	0.35	314	92		-22.0	2.7			56

ID	Site	Species	Description	%C	%N	C/N	%S	C:S	N:S	δ^{34}S	δ^{13}C	δ^{15}N	^{14}C Age (B.P.)	Ref. for dates	Ref. for δ^{13}C and δ^{15}N
SC3100	Scladina	*Ursus spelaeus*	Mandible	40.9	14.8	3.2	0.15	727	226	0.1	-22.5	3.7			56
SC3200	Scladina	*Ursus spelaeus*	Mandible	42.4	15.2	3.3					-22.1	5.7			56
SC3300	Scladina	*Ursus spelaeus*	Mandible	43.3	15.8	3.2	0.16	709	222	-2.1	-22.2	6.0			56
SC3500	Scladina	*Ursus spelaeus*	Phalanx II	44.0	16.0	3.2	0.16	731	228	3.4	-21.8	5.1			56
SC3600	Scladina	*Ursus spelaeus*	Phalanx II	42.8	15.6	3.2	0.16	722	225	-17.0	-21.8	3.0			56
SC3700	Scladina	*Ursus spelaeus*	Phalanx II	42.4	15.4	3.2	0.16	699	218	-5.4	-22.0	6.1			56
SC3800	Scladina	*Ursus spelaeus*	Phalanx II	42.5	15.5	3.2	0.15	732	229	-4.2	-22.2	5.0			56
Goyet-A2-1	Goyet	*Mammuthus primigenius*	Long bone	41.0	15.0	3.2	0.13	846	265	-2.6	-20.7	8.1			56
Goyet-A3-9	Goyet	*Mammuthus primigenius*	Long bone	43.9	15.6	3.3	0.16	732	223	4.1	-21.5	7.0			56
Goyet-B4-2	Goyet	*Mammuthus primigenius*	Skull	41.0	14.6	3.3	0.15	730	223	8.4	-21.6	6.7			56
SC600	Scladina	*Mammuthus primigenius*	Tooth frag.	42.6	15.5	3.2	0.14	830	259	6.0	-20.9	8.4			50
SC700	Scladina	*Mammuthus primigenius*	Tooth frag.	41.5	15.2	3.2	0.13	828	260	6.6	-21.5	9.4			50
SC800	Scladina	*Mammuthus primigenius*	Tooth frag.	41.3	15.1	3.2	0.14	796	249	4.5	-21.6	8.3			50
Spy D3 19B 121 1474	Spy	*Mammuthus primigenius*	Milk molar	35.1	12.50	3.3					-21.1	11.6	42330 +550/-450 (GrA-32616)	51	52
IV2A 13549	Spy	*Mammuthus primigenius*	Plate of molar	37.7	13.8	3.2					-21.3	8.1	42950 +800/-650 (GrA-44548)	55	57
Goyet-A2-2	Goyet	*Coelodonta antiquitatis*	Unciform	43.8	16.2	3.2					-20.0	4.3			56
Goyet-A3-2	Goyet	*Coelodonta antiquitatis*	Scaphoid	43.8	15.2	3.4	0.18	647	193	-0.1	-20.4	5.7			56

ID	Site	Species	Description	%C	%N	C/N	%S	C:S	N:S	$\delta^{34}S$	$\delta^{13}C$	$\delta^{15}N$	^{14}C Age (B.P.)	Ref. for dates	Ref. for $\delta^{13}C$ and $\delta^{15}N$
Goyet-B4-3	Goyet	*Coelodonta antiquitatis*	1st metacarpal	40.0	14.6	3.2					-20.8	5.9			56
SC30100	Scladina	*Coelodonta antiquitatis*	Rt metatarsal II	44.4	15.7	3.3					-21.1	5.5			56
SC900	Scladina	*Cœlodonta antiquitatis*	Lower rt P2	42.6	15.5	3.2	0.13	874	273	6.9	-20.9	5.5			50
SC1000	Scladina	*Cœlodonta antiquitatis*	Lower rt P2	42.1	15.4	3.2	0.13	872	274	1.9	-20.3	6.9			50
SC1100	Scladina	*Cœlodonta antiquitatis*	Lower rt P2	42.2	15.2	3.2	0.14	822	254	4.6	-20.0	6.4			50
SC1200	Scladina	*Cœlodonta antiquitatis*	Lower rt P2	41.2	15.0	3.2	0.14	806	251	2.5	-21.1	5.3			50
SC1300	Scladina	*Cœlodonta antiquitatis*	Lower rt P2	41.8	15.2	3.2	0.12	900	281		-20.4	7.5			50
SC1400	Scladina	*Cœlodonta antiquitatis*	Lower rt P2	43.1	15.7	3.2					-20.6	5.5			50
Spy 13637 ULg	Spy	*Cœlodonta antiquitatis*	Lower P3 or P4	43.3	14.2	3.6					-20.8	6.8	25670 +130/-120 (GrA-37936)		57
Spy D2 Pal Plateau 4	Spy	*Cœlodonta antiquitatis*	Milk molar	40.4	14.4	3.3					-20.1	7.2	44350 +650/-500 (GrA-32613)	55	52
Goyet-A3-10	Goyet	*Equus ferus*	Ectocuneiform	44.9	15.9	3.3	0.15	822	249	0.6	-21.0	5.1			56
Goyet-A3-11	Goyet	*Equus ferus*	Ectocuneiform	41.9	15.4	3.2	0.14	800	252	1.0	-20.7	6.4			56
Goyet-A3-12	Goyet	*Equus ferus*	Ectocuneiform	43.9	15.4	3.3	0.16	746	224	-0.7	-20.8	5.9			56
Goyet-B4-4	Goyet	*Equus ferus*	Tibia	41.0	14.8	3.2					-20.5	6.6			56
SC28400	Scladina	*Equus ferus*	Upper lt P2	34	12.9	3					-21.1	6.5			56
SC3900	Scladina	*Equus ferus*	Upper rt tooth	42.5	15.6	3.2	0.14	790	248	5.7	-21.7	5.2			50
SC4100	Scladina	*Equus ferus*	Upper rt tooth	42.6	15.8	3.1	0.13	908	289		-21.7	5.1			50
SC4200	Scladina	*Equus ferus*	Upper rt tooth	42.8	15.8	3.2	0.13	874	276	2.0	-21.9	5.0			50
SC4300	Scladina	*Equus ferus*	Upper rt tooth	39.9	14.5	3.2	0.14	781	244	6.2	-21.5	4.8			50
SC4400	Scladina	*Equus ferus*	Upper rt tooth	40.9	15.0	3.2	0.12	910	286		-21.6	7.0			50

ID	Site	Species	Description	%C	%N	C/N	%S	C:S	N:S	δ^{34}S	δ^{13}C	δ^{15}N	^{14}C Age (B.P.)	Ref. for dates	Ref. for δ^{13}C and δ^{15}N
IV2E 4207	Spy	*Equus ferus*	Premolar / Molar	35.6	14.2	2.9	0.15	633	216	5.5	-20.5	4.5	32810 +250/-230 (GrA-44576)	55	57
Goyet-B4-6	Goyet	*Bos primigenius*	Centrotarsus	38.7	14.1	3.2					-20.0	3.8			56
Goyet-A3-13	Goyet	*Bison priscus*	Tibia	40.9	14.6	3.3	0.16	687	209	-2.8	-20.0	4.1			56
Goyet-A3-14	Goyet	*Bison priscus*	Tibia	41.4	14.6	3.3	0.15	725	219	4.9	-20.4	4.8			56
Goyet-B4-5	Goyet	*Bison priscus*	Centrotarsus	40.9	14.7	3.2					-20.0	5.7			56
Goyet-B4-7	Goyet	*Bison priscus*	Centrotarsus	38.8	14.1	3.2					-20.4	3.9			56
SC29000	Scladina	*Bison priscus*	Lower rt M3	32.6	11.5	3.3					-20.8	4.4			56
SC29100	Scladina	*Bison priscus*	Lower lt P4	32.1	12.8	2.9					-19.8	5.6			56
SC4500	Scladina	*Bos or Bison*	Lower rt P	41.7	15.2	3.2	0.15	764	239	4.1	-20.5	4.8			50
SC4700	Scladina	*Bos or Bison*	Lower rt P3	42.7	15.7	3.2	0.14	806	254	8.4	-20.5	4.3			50
SC4800	Scladina	*Bos or Bison*	Lower rt M1	41.2	15.3	3.1					-19.9	5.3			50
SC4900	Scladina	*Bos or Bison*	Lower rt P3	40.8	15.0	3.2	0.15	725	229	4.5	-20.7	4.4			50
SC28800	Scladina	*Bos primigenius*	Lower lt M3	33.9	12.7	3.1					-20.7	4.9			56
SC28900	Scladina	*Bos primigenius*	Lower lt P4	35.0	13.6	3.0					-20.3	4.5			56
Goyet-A3-15	Goyet	*Rangifer tarandus*	Astragalus	42.3	15.5	3.2	0.17	667	209	7.2	-18.5	2.6			56
Goyet-A3-16	Goyet	*Rangifer tarandus*	Astragalus	43.0	15.1	3.3	0.18	620	187	6.2	-18.5	3.3			56
Goyet-A3-17	Goyet	*Rangifer tarandus*	Astragalus	43.4	15.3	3.3	0.14	803	242	5.1	-18.6	3.4			56
Goyet-A3-18	Goyet	*Rangifer tarandus*	Astragalus	43.9	15.4	3.3	0.15	773	232	6.8	-18.3	3.1			56
Goyet-A3-19	Goyet	*Rangifer tarandus*	Astragalus	44.1	15.2	3.4	0.15	787	232	-7.2	-19.6	4.5			56
Goyet-B4-8	Goyet	*Rangifer tarandus*	Centrotarsus	41.1	14.9	3.2					-18.6	3.9			56

ID	Site	Species	Description	%C	%N	C/N	%S	C:S	N:S	$\delta^{34}S$	$\delta^{13}C$	$\delta^{15}N$	^{14}C Age (B.P.)	Ref. for dates	Ref. for $\delta^{13}C$ and $\delta^{15}N$
Spy 10640 Ulg	Spy	*Rangifer tarandus*	Phalanx I	43.4	14.4	3.5					-19.8	4.9	29040 +180/-160 (GrA-37934)	51	56
Spy 13071 Ulg	Spy	*Rangifer tarandus*	Metacarpal	35.6	14.1	2.9					-18.4	1.6	36920 +400/-350 (GrA-44546)	55	56
Spy D4 19B 121 1480	Spy	*Rangifer tarandus*	Metacarpal	39.3	17.4	2.6	0.14	735	279						51
SC29700	Scladina	*Rangifer tarandus*	Tooth	37.5	12.4	3.5	0.2	625	178	8.3	-17.9	3.5			this study
Sc 22400	Scladina	*Rangifer tarandus*	P2-P4	35.6	12.9	3.2	0.13	746	232	7.49	-18.7	3.1			this study
Sc 22700	Scladina	*Rangifer tarandus*	Lower M2	38.8	14.2	3.2	0.14	761	239	8.49	-18.5	5.0			this study
Sc 22800	Scladina	*Rangifer tarandus*	Lower M1	39.6	14.5	3.2	0.15	706	221	10.52	-17.6	3.6			this study
Sc 22900	Scladina	*Rangifer tarandus*	Upper P4	39.7	14.6	3.2	0.15	696	219	6.48	-17.8	4.8			this study
Sc 23000	Scladina	*Rangifer tarandus*	Upper M2	40.9	14.9	3.2	0.14	784	245	13.13	-16.7	7.2			this study

Abbreviations: Rt: right; Lt: left; frag.: fragment.
$\delta^{34}S$ values are all from this study. Isotopic values in red are not considered due to preservation issues. All isotopic values and indicated chemical parameters refer to bone collagen.

Supplementary Table S3-List of stable isotopic data from herbivorous and carnivorous mammal species from the Aurignacian open-air site of Lommersum

ID	Excav. No.	Species	Description	%C	%N	C:N	%S	C:S	N:S	$\delta^{34}S$	$\delta^{13}C$	$\delta^{15}N$
Lom-1	A12 67	*Equus ferus*	Rt radius	11.67	5.02	2.71					-21.18	1.70
Lom-2	E5 113	*Rangifer tarandus*	Humerus	41.88	15.34	3.18	0.23	488.60	153.45	1.43	-19.14	3.70
Lom-3	E4 690	*Rangifer tarandus*	Rt radius	44.48	16.05	3.23	0.21	553.51	171.17	4.20	-19.19	3.78
Lom-4	E6 196	*Rangifer tarandus*	Rt radius	39.37	14.38	3.19	0.20	537.13	168.16	4.73	-18.95	3.19
Lom-5	E5 523	*Rangifer tarandus*	Rt dist. radius	40.00	14.00	3.30	0.28	383.08	114.92	6.92	-19.40	3.30
Lom-6	F4 142	*Rangifer tarandus*	Rt dist. radius	43.51	15.73	3.23	0.20	593.53	183.89	2.57	-18.35	3.30
Lom-7	X12 82	*Rangifer tarandus*	Rt dist. radius	18.20	6.80	3.10	0.38	127.05	40.69	8.96	-19.90	2.00
Lom-8	B5 265	*Rangifer tarandus*	Rt dist. radius	45.18	16.43	3.21	0.18	675.90	210.73	5.59	-18.33	3.43
Lom-9	C6 532	*Rangifer tarandus*	Rt dist. radius	38.28	14.06	3.18	0.27	371.41	116.90	6.15	-18.79	3.60
Lom-10	D5 273	*Rangifer tarandus*	Rt dist. radius	39.35	14.29	3.21	0.32	332.90	103.61	6.96	-18.97	2.97
Lom-11	C7 12	*Rangifer tarandus*	Rt dist. radius									
Lom-12	D4 161	*Equus ferus*	Rt radius	43.97	15.87	3.23	0.16	723.99	223.92	2.00	-20.62	6.64
Lom-13	C4 292	*Equus ferus*	Rt med. radius	41.80	15.37	3.17	0.20	555.17	174.93	3.45	-20.68	7.31
Lom-14	C6 622	*Canis lupus*	Dist. tibia	35.38	12.46	3.31					-20.23	7.68
Lom-15	E4 351	*Panthera leo*	Incisor	38.00	13.50	3.30					-19.20	8.50
Lom-16	C5 158	*Equus ferus*	Dist. tibia	38.77	13.68	3.31					-20.88	4.21
Lom-17	Z10 31	*Equus ferus*	Dist. tibia									

ID	Excav. No.	Species	Description	%C	%N	C:N	%S	C:S	N:S	$\delta^{34}S$	$\delta^{13}C$	$\delta^{15}N$
Lom-18	D7 99	*Equus ferus*	Dist. tibia									
Lom-19	LO B5 IIc	*Mammuthus primigenius*	Ivory	27.05	9.10	3.47					-20.64	7.57
Lom-20	Lo D6 IIc2 327	*Equus ferus*	Rt humerus	39.20	13.78	3.32					-20.94	4.97
Lom-21	Lo E5 IIC 127	*Equus ferus*	Rt radius	42.38	14.87	3.33					-20.71	6.30
Lom-22	LO F5 IIb 183	*Equus ferus*	Rt humerus	40.94	14.46	3.30					-20.41	4.51
Lom-23	LO C7 IIc 154	*Equus ferus*	Rt? radius	No collaqgen preserved								

Abbreviations: Excav. No.: excavation number; Rt: right; Lt: left; dist.: distal; med.: medial.
All of the stable isotopic values are from this study. Values in red are not considered due to preservation issues.

Supplementary Table S4-Herbivore assemblages from the Troisième caverne of Goyet and Spy

Herbivore assemblage	Goyet A1-A2-A3-B4		Spy	
	NISP	%	NISP	%
Mammuthus primigenius	200	4.37	1178	15.89
Equus sp.	1968	43.04	4534	61.15
Coelodonta antiquitatis	405	8.86	620	8.36
Sus scrofa	4	0.09	5	0.07
Cervus elaphus	100	2.19	198	2.67
Capreolus capreolus	10	0.22		
Megaloceros giganteus	3	0.07	11	0.15
Rangifer tarandus	1666	36.43	392	5.29
Bison priscus/Bos primigenius	142	3.11	468	6.31
Ovibos moschatus	8	0.17		
Rupicapra/Capra	67	1.47	9	0.12
Total	4573		7415	

Supplementary Table S5-Mammoth assemblage from the Troisième caverne of Goyet

Goyet, cave 3, Total	
Mammuthus	**NISP**
Cranium	4
Maxilla	5
Mandible	1
Ivory	120
Molars	28
M1	*1*
M2	*4*
M3	*5*
M4	*5*
M5	*2*
M frag.	*11*
Rib	7
Scapula/ilium	1
Humerus	2
Femur	2
Diaphysis	30
Total	200

Supplementary Table S6-Age distribution of the mammoth molars from the Troisième caverne of Goyet and Spy

Mammoth molars		Goyet	
Laws' (1966) age classes	NISP	%NISP	MNI
I-III (≤ 4 a.e.y.)	9	52.94	3
VII-X (5-12 a.e.y.)	4	23.53	2
XI-XVI (13-24 a.e.y.)	3	17.65	2
XVIII-XXII (> 24 a.e.y.)	1	5.88	1
Total	17		

Mammoth molars	Spy	
Laws' (1966) age classes	NISP	%NISP
I-IV (≤ 4 a.e.y.)	32	59.26
VII-IX (5-12 a.e.y.)	9	16.67
XII-XVII (13-24 a.e.y.)	9	16.67
XVIII-XXVII (> 24 a.e.y.)	4	7.41
Total	54	

Supplementary Figure 1-Cluster analysis of Late Pleistocene herbivorous species from the Troisième caverne of Goyet, Scladina and Spy based on $\delta^{15}N$ and $\delta^{13}C$ bone collagen values

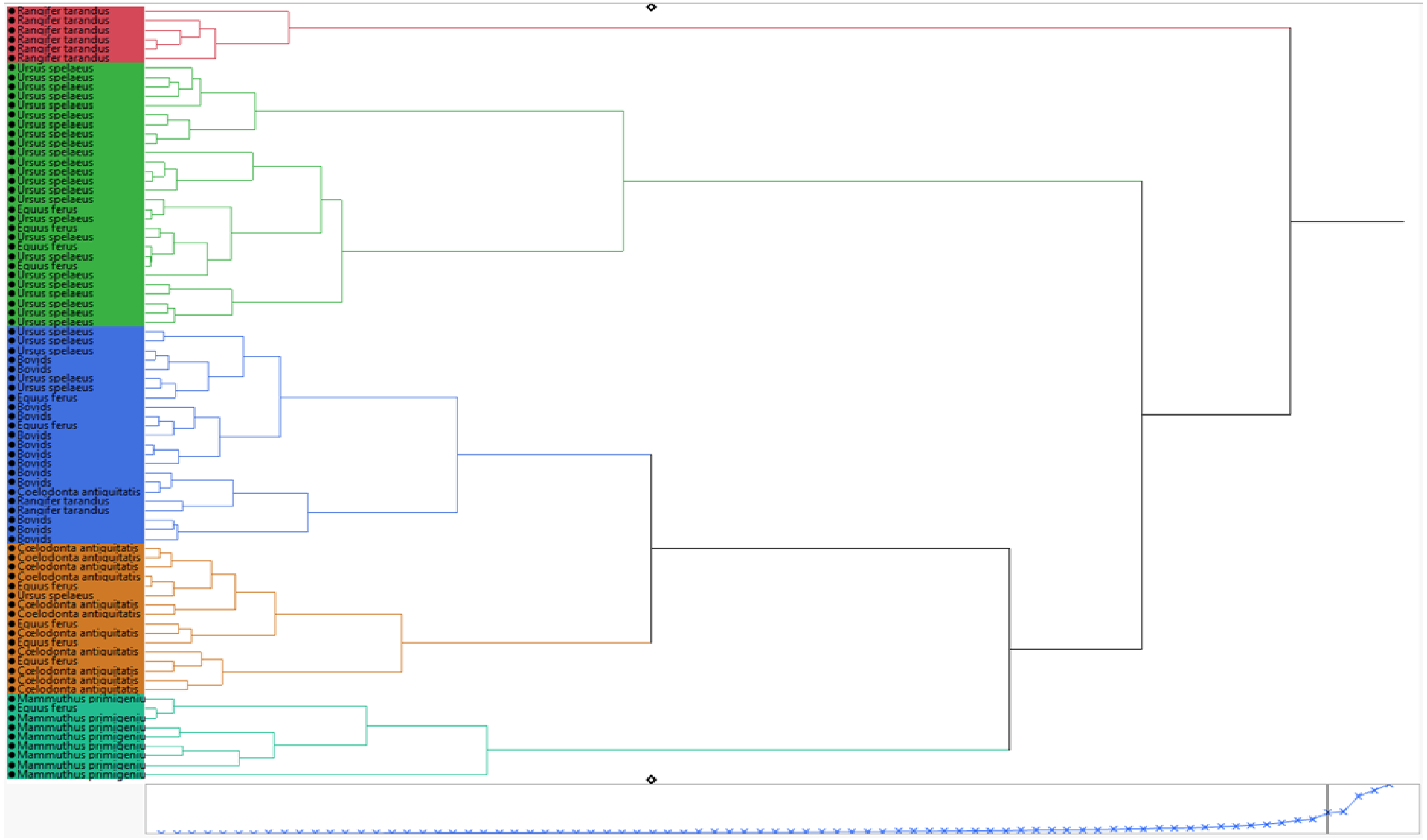

Analysis performed with the software SAS JMP version 10.0.

Supplementary Figure 2-Proportion vs. density plots showing the probability distributions of the amount of different protein sources (species) in the diet of the Belgian UPMHs

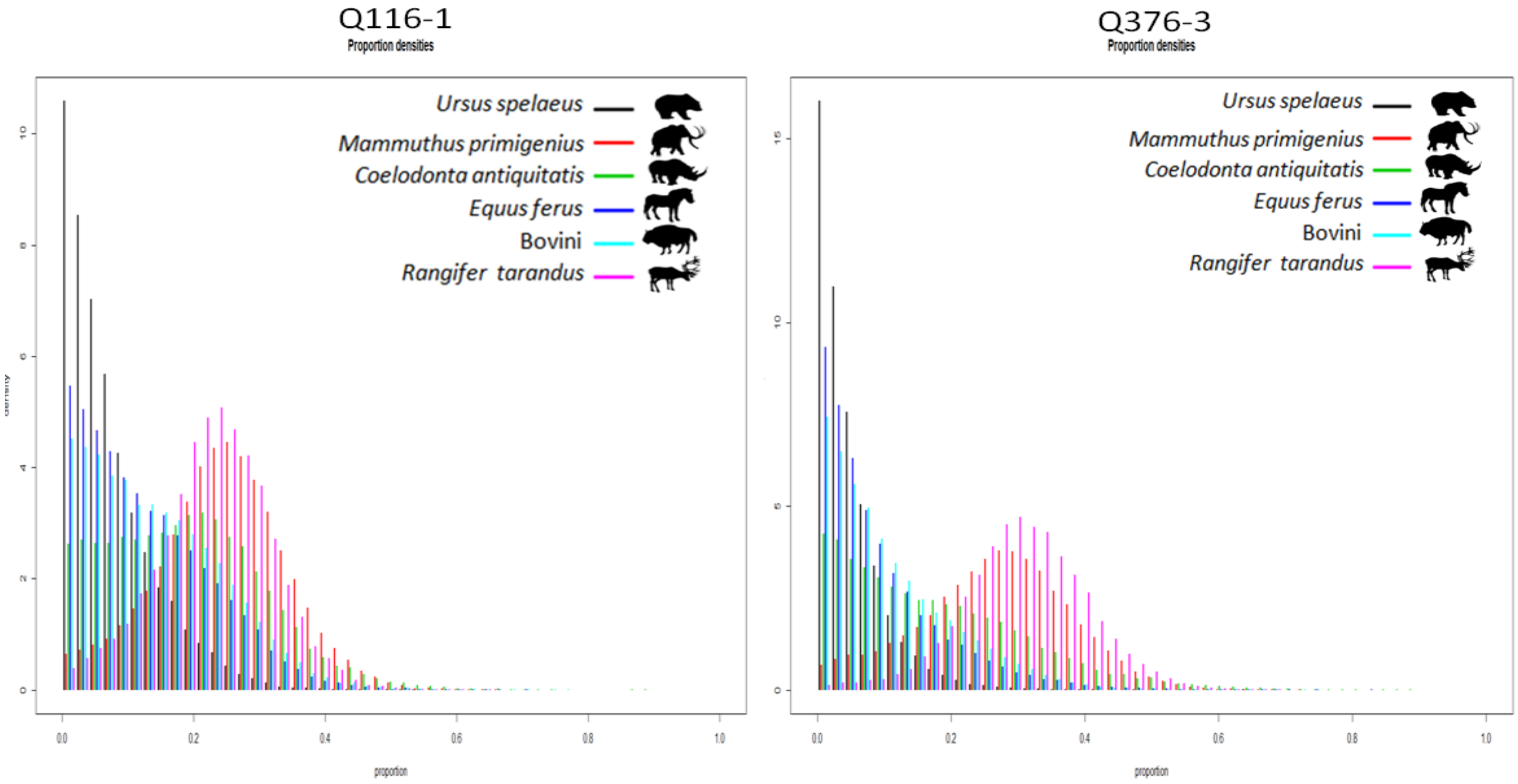

Specific amounts of different prey species in the diet of the UPMHs from Goyet (i.e. proportions between 0 % and 100 %) are plotted on the x-axis in regard to their probabilities (i.e. densities between 0 % and 100 %) on the y-axis. The higher the proportion, the higher the relative contribution of the protein source and vice versa. The higher the density of a given amount, the higher the probability for this specific amount and vice versa. Calculations were performed through the application of a Bayesian method (SIAR V4, Stable Isotope Analysis-package in R)[58,59].

Supplementary Figure 3-Diagnostic matrix plots summarizing the statistical dependencies between different protein sources for the UPMHs from the Troisième caverne of Goyet

Q116-1

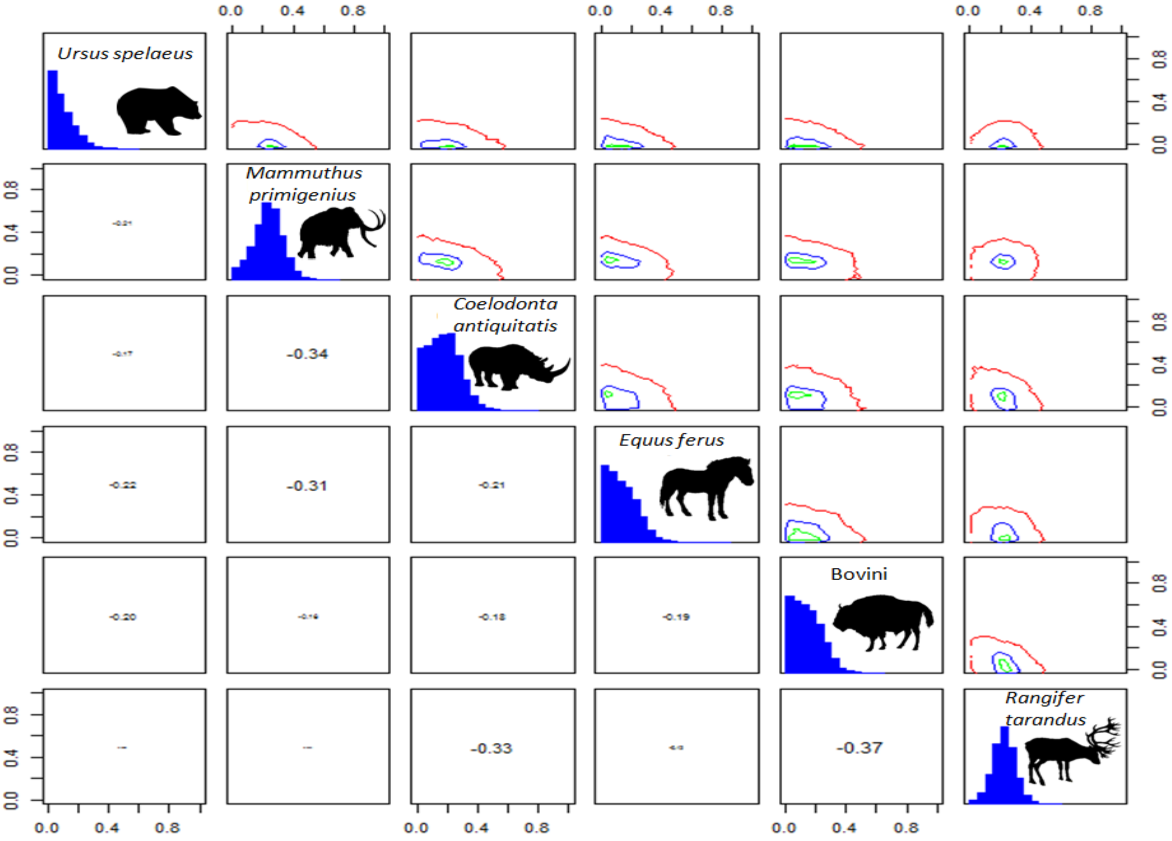

Q376-3

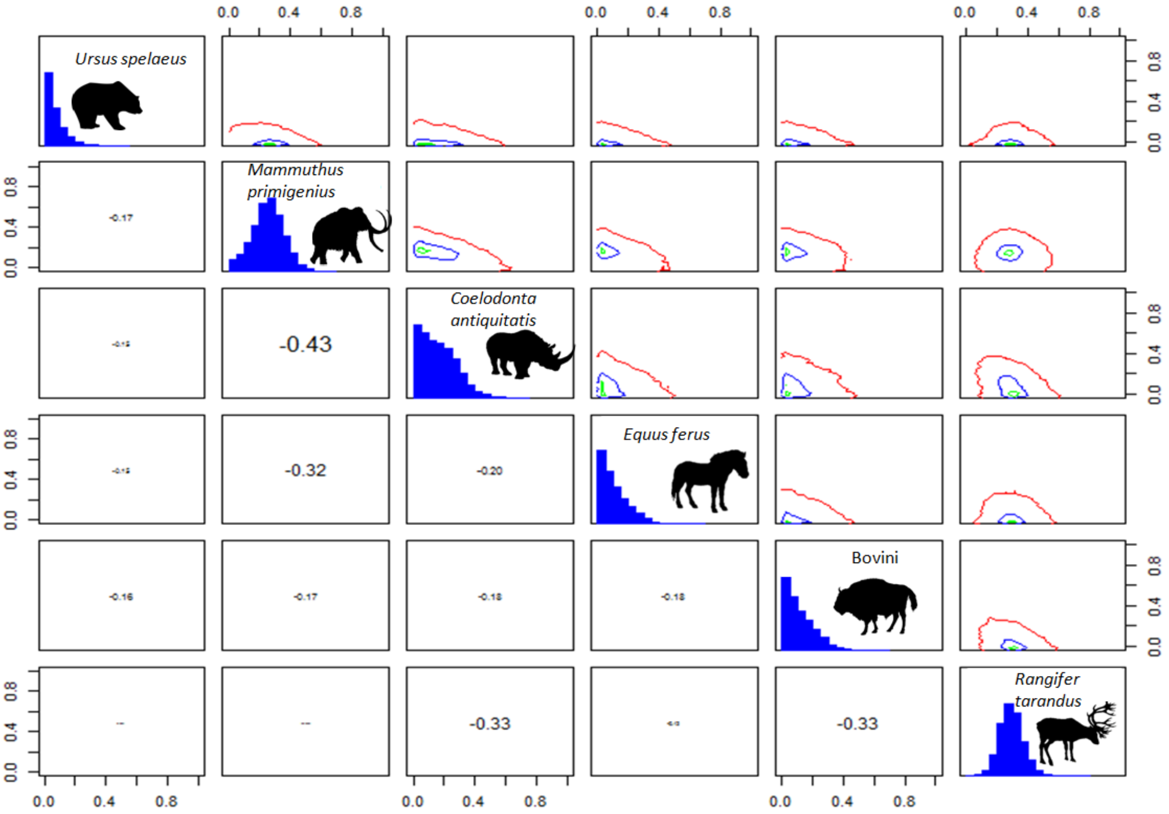

Dependencies can be either negative or positive. Calculations were performed through the application of a Bayesian method (SIAR V4, Stable Isotope Analysis-package in R)[58,59]. The SIAR software takes into account the correlations among different sources (prey species), which end up in an increasing probability range. The absolute values of the numbers specify the intensity of the correlations. Correlations indicate that distinct species can be at least partly replaced by other ones, either by one species or by mixing two or more to end up in the range of the isotopic signatures of the original one.

Supplementary Figure 4-Age groups of the mammoths from Goyet and Spy

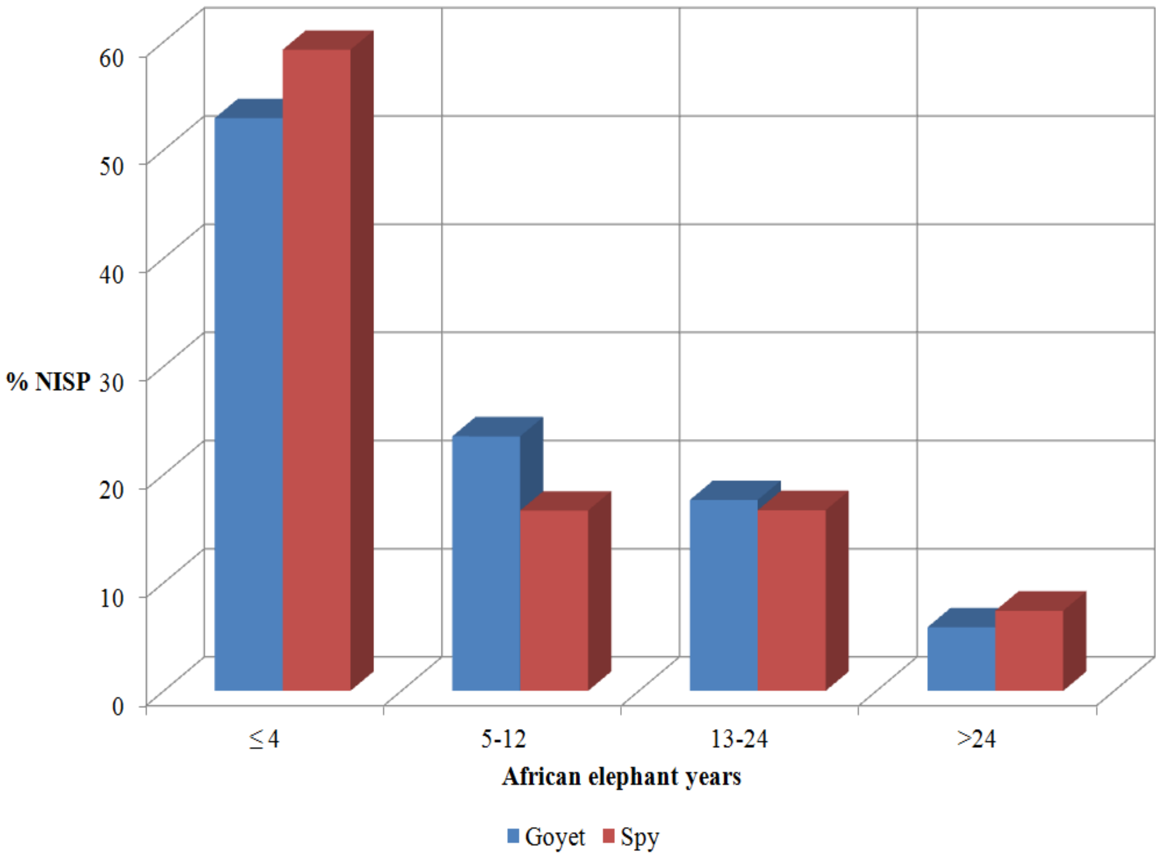

Supplementary Figure 5-Box plots of the relative contributions of different prey species in the diet of the Saint-Césaire I Neandertal (France)

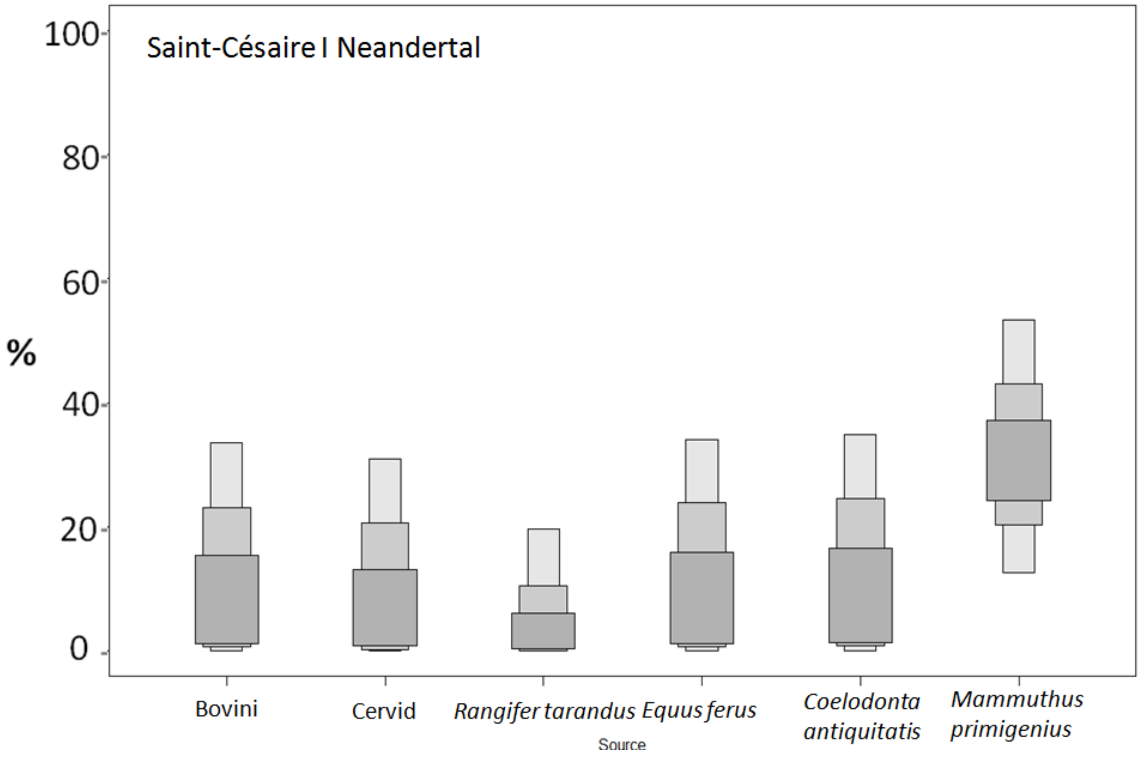

Calculations were performed through the application of a Bayesian method (SIAR V4, Stable Isotope Analysis-package in R)[58,59]. Within the proportion box plots, three different grey shades are shown: the lightest grey represents a probability of 95 %, the medium grey 75 %, and the darkest grey 25 %.

Supplementary Figure 6-Diagnostic matrix plots summarizing the statistical dependencies between different protein sources for the Saint-Césaire I Neandertal (France)

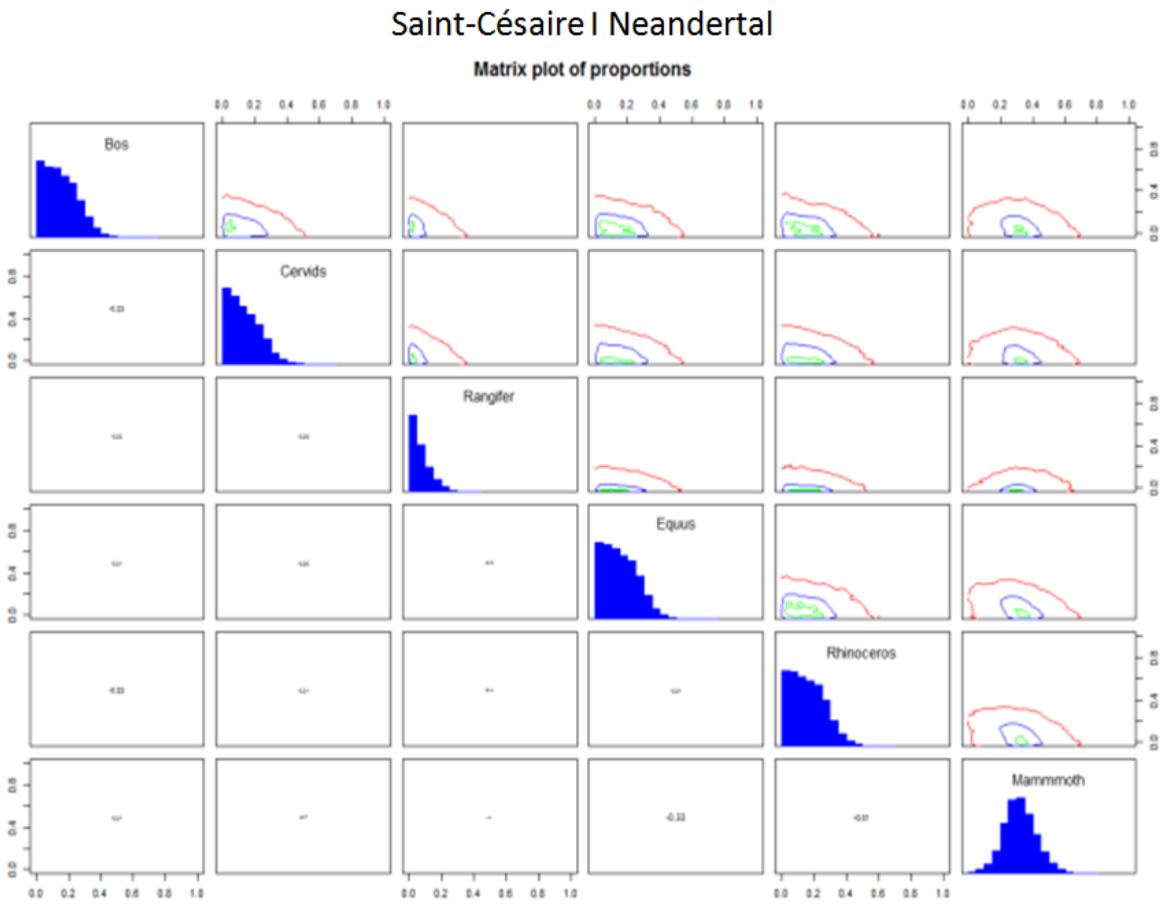

Dependencies can be either negative or positive. Calculations were performed through the application of a Bayesian method (SIAR V4, Stable Isotope Analysis-package in R)[58,59]. The SIAR software takes into account the correlations among different sources (prey species), which end up in an increasing probability range. The absolute values of the numbers specify the intensity of correlations. Correlations indicate that distinct species can be at least partly replaced by other ones, either by one species or by mixing two or more to end up in the range of the isotopic signatures of the original one.

Supplementary Figure 7-Proportion vs. density plot showing the probability distributions of the amount of different protein sources (species) in the diet of the Saint-Césaire I Neandertal (France)

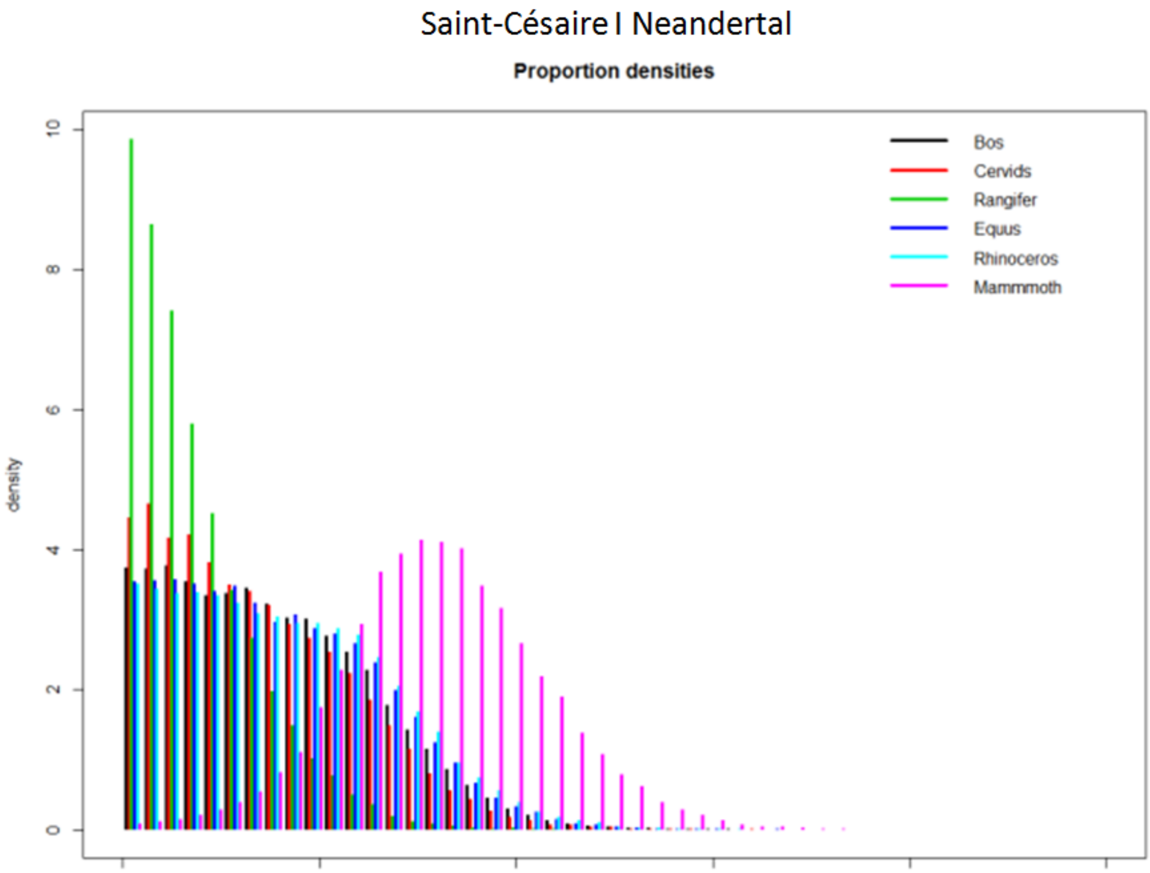

Specific amounts of different prey species in the diet of the Saint-Césaire I Neandertal (i.e. proportions between 0 % and 100 %) are plotted on the x-axis in regard to their probabilities (i.e. densities between 0 % and 100 %) on the y-axis. The higher the proportion, the higher the relative contribution of the protein source and vice versa. The higher the density of a given amount, the higher the probability for this specific amount and vice versa. Calculations were performed through the application of a Bayesian method (SIAR V4, Stable Isotope Analysis-package in R)[58,59].

Supplementary Figure 8-Box plots of the relative contribution of mammoth meat for different large predators

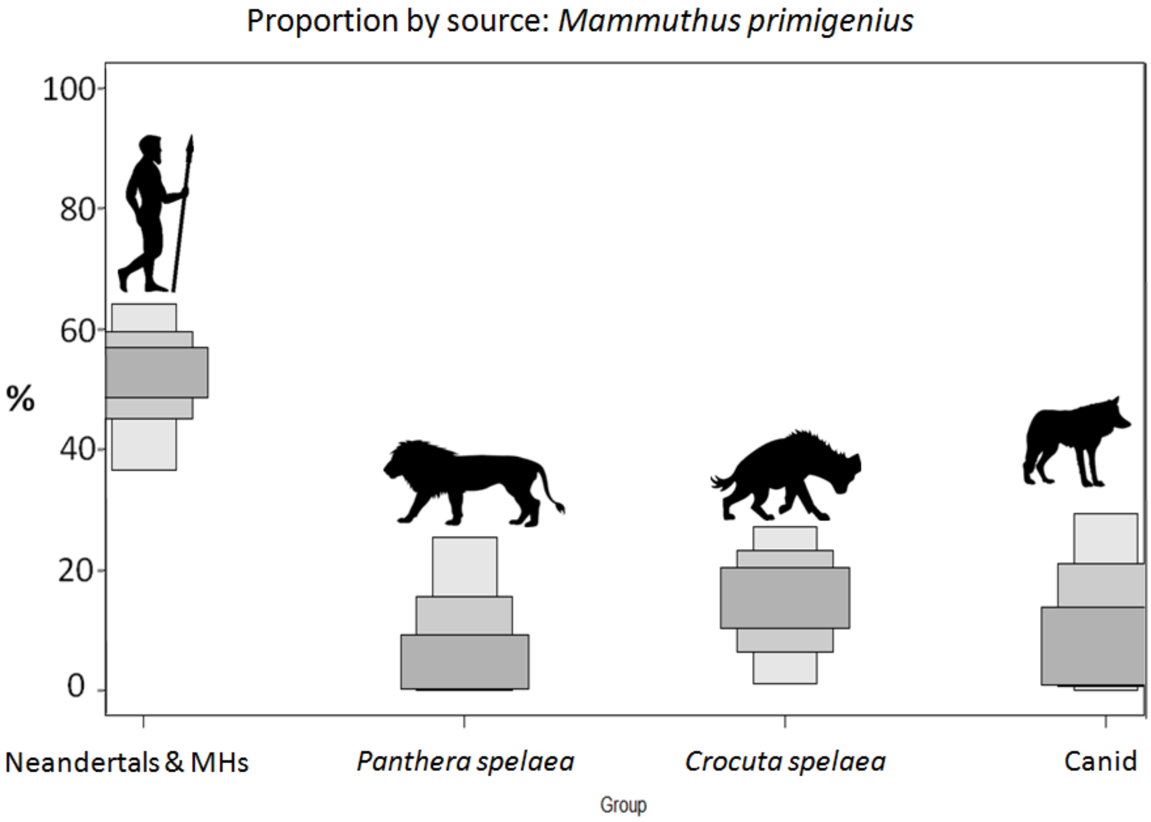

The large predators are from left to right: humans, cave lion, hyena, and wolf. Values from Neandertals (Goyet, Spy) and UPMHs (Q116-1, Q376-3) are combined. Calculations were performed through the application of a Bayesian method (SIAR V4, Stable Isotope Analysis-package in R)[58,59]. Within the proportion box plots, three different grey shades are shown: the lightest grey represents a probability of 95 %, the medium grey 75 %, and the darkest grey 25 %.

Supplementary Figure 9-Horse and mammoth carbon and nitrogen stable isotopic data presented in chronological order

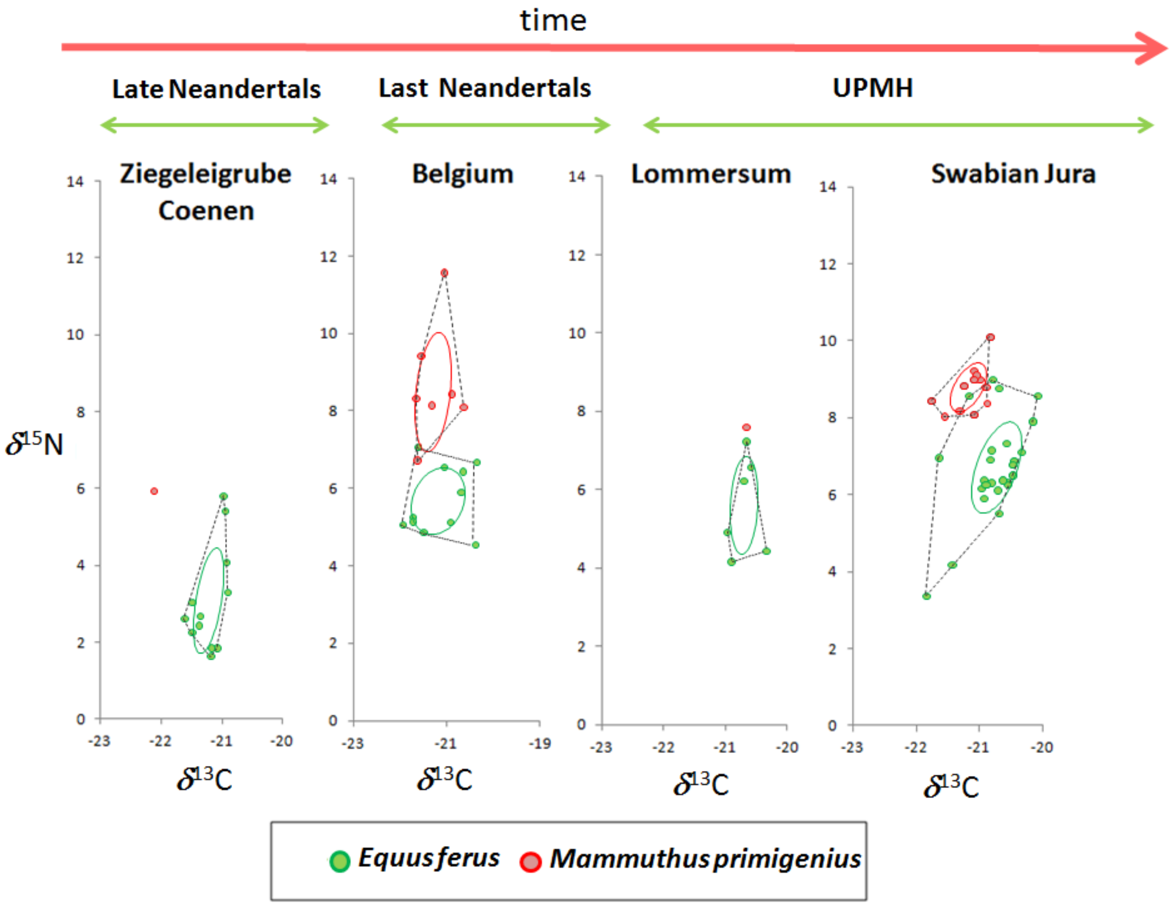

The complete niches/convex hulls (dashed lines) and the core niches (solid lines) of mammoth and horse specimens are presented in chronological order. During Neandertal times, the ecological niche separation of horse and mammoth was relatively stable. However, with the onset of the Upper Palaeolithic, single horse individuals yield values similar to mammoth values, and a partial overlap of their niches can be tracked (Supplementary Data 8). This is most probably due to a partially vacant mammoth niche as mammoth was a key prey species.

Supplementary Figure10-Upper first milk molar of a juvenile mammoth (2777-6) from the Troisième caverne of Goyet, Belgium (Dupont collection, Royal Belgian Institute of Natural Sciences). Photo credit: Royal Belgian Institute of Natural Sciences

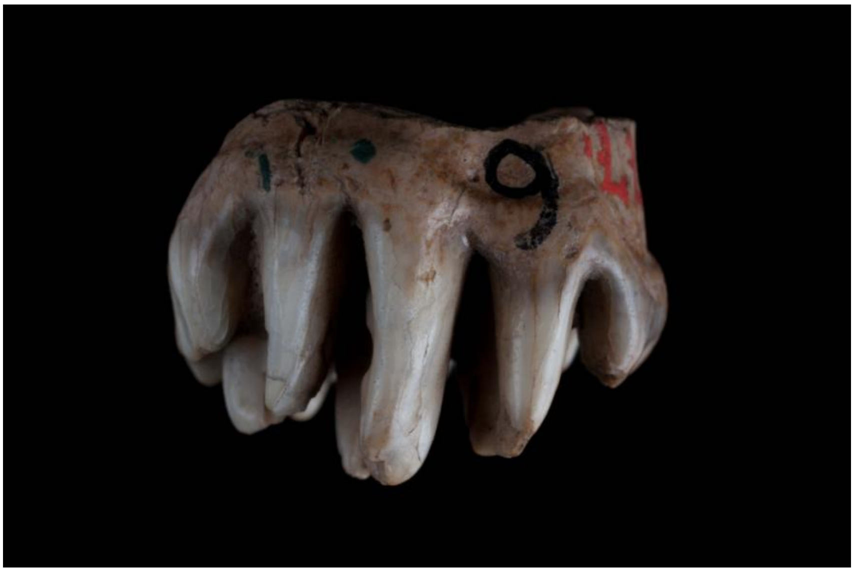

Length: 17.83 mm, width: 15.14 mm; specimen ID: 2777-6.
